# Supplementary material for: Improved Quantitative Real-Time PCR Protocol for Detection and Quantification of Methanogenic Archaea in Stool Samples
Source: Microorganisms. 2023 Mar 5;11(3):660. doi: 10.3390/microorganisms11030660 (PMC10051802; doi:10.3390/microorganisms11030660)
Supplement: Supplementary file 1 [file microorganisms-11-00660-s001.zip › Table S1.pdf]

```
CLUSTAL

Methanopyrus_sp._18_510_KC618341.1/1-477 GGTGGAGT-CGGATT-CACACAGTACGCAACAGCAGCATACACCGATAACGTCCTGGAAG
Methanomassiliicoccus_luminyensis_1_456_strain_B10_HQ896500.1/1-455 GGTGGTGT-AGGATT-CACACAGTATGCTACAGCGGCCTACACCGACGACATCCTGGAGG
Methanopyrus_kandleri_1_414_AF414042.1/1-414 --------------------------------------TACACTGATAACATCCTGGACG
Methanosarcina_mazei_MH004454.1_RCompl_1_433/1-433 ----------------------------------TGCATACACAGACGACATCCTCGGCA
Methanosarcina_horonobensis_256_737_AB288266.1/1-466 GGTGGTGT-CGGGTT-CACACAGTATGCAACAGCTGCATACACTGATGACATCCTCGACA
Methanosarcina_subterranea_259_740_AB288268.1/1-466 GGTGGTGT-CGGGTT-CACACAGTATGCAACAGCTGCATACACTGATGACATCCTCGACA
Methanolobus_oregonensis_1_490_U22242.1/1-474 GGTGGTGT-AGGTTT-CACACAGTACGCTACAGCAGGTTACACCAACAACATCCTTGATG
Methanolobus_zinderi_256_745_EU715818.1_RCompl/1-474 GGTGGTGT-AGGTTT-CACCCAGTACGCAACAGCAGCATACTGTAACAACATCCTTGATG
Methanolobus_taylorii_1_490_U22243.1/1-474 GGTGGTGT-CGGCTT-CACCCAATACGCTACAGCAGCATACTGTAACAACATCCTTGATG
Methanolobus_profundi_687_1168_AB703629.1/1-466 GGTGGTGT-AGGTTT-CACCCAGTACGCAACAGCAGCATACTGTAACAACATCCTTGATG
Methanolobus_vulcani_1_490_U22245.1/1-474 GGTGGTGT-AGGTTT-CACCCAGTACGCAACAGCAGCATACTGTAACAACATCCTTGACG
Methanocella_conradii_1_465_JN081865.1/1-450 GGTGGTGT-GGGCTT-CACGCAGTACGCGACCGCCGCATACACCGATGACATCCTGGATG
Methanocella_sp._279_692_KJ441441.1/1-398 GGTGGTGT-CGGCTT-CACGCAGTATGCGACCGCCGCCTACACCGATGACATCCTGGATG
Methanocella_paludicola_240_681_AB300467.1/1-426 GGTGGTGT-CGGCTT-CACGCAGTATGCGACCGCCGCCTACACCGATGATATCCTGGATG
Methanomicrobium_mobile_1_438_AF414044.1/1-438 --------------------------------------TACACGGATAACATCCTTGATG
Methanoculleus_marisnigri_RCompl_1_436_MH004450.1/1-436 ----------------------------------GGCCTACACCGACAACATCCTCGATG
Methanoculleus_palmolei_259_743_AB300784.1/1-469 GGCGGTGT-CGGCTT-CACGCAGTATGCGACTGCAGCCTACACCGACAACATCCTCGATG
Methanoculleus_bourgensis_259_743_AB300787.1/1-469 GGCGGTGT-CGGATT-CACCCAGTACGCGACCGCGGCCTACACCGACAACATCCTCGATG
Methanoculleus_chikugoensis_259_743_AB300779.1/1-469 GGCGGTGT-CGGATT-CACCCAGTACGCAACCGCGGCCTACACCGACAACATCCTCGACG
Methanogenium_organophilum_1_501_DQ229160.1/1-487 GGTGGTGT-CGG-TT-CACCCAGTATGCTACTGCAGCATACACCGATGACATCCTCGATG
Methanogenium_boonei_15_520_DQ229161.1/1-490 GGTGGTGT-CGG-TT-CACGCAGTATGCAACAGCAGCATACACCGATAACATCCTCGATG
Methanogenium_cariaci_1_433_DQ229157.1/1-420 GGTGGTGT-CGG-TTTCACCCAGTATGCAACCGCAGCATACACCGAT-ACATCCTTGATG
Methanobacterium_sp._1_452_JQ917190.1/1-452 -------------TT-CACCCAGTATGCAACCGCAGCTTACACTGACGATATTCTGGACG
Methanobrevibacter_smithii_1_423_GU385700.1/1-423 TGTGGTGTTCGGATT-CACACAGTATGCAACAGCAGCATATACTGATGATGTATTAGATG
Methanobrevibacter_oralis_1_414_LK054628.1/1-414 -----------------------------------GCATATACTGATAATGTATTAGATG
Methanobrevibacter_ruminantium/1-470 GGTGGTGT-AGGATT-TACTCAATATGCTACCGCAGCATACACCGATAACGTATTAGACG
Methanobrevibacter_sp._D5_mcrA_plasmid_365_874_KF214818.1/1-488 GGTGGTGT-AGGATT-CACACAATATGCTACTGCAGCATACACTGATAATGTATTAGACG
Methanobrevibacter_gottschalkii_1_471_EU919431.1/1-471 GGTGGTGT-AGGATT-CACACAGTATGCAACAGCTGCATACACAGATAATGTATTAGATG
Methanobrevibacter_boviskoreani_1_424_KC865050.1/1-424 --------------------------------------------------CTTCTTGATG
Methanobrevibacter_woesei_1_467_EU919432.1/1-467 GGTGGTGT-AGGATT-CACACAGTATGCTACAGCAGCATATACTGATAATGTTCTTGATG
Methanobrevibacter_wolinii_1_395_KC865051.1/1-395 ------------------------------------------------------------
Methanobrevibacter_arboriphilus_1_414_AF414035.1/1-414 --------------------------------------TACACTGACAATATTCTGGACG
Methanobacterium_movens_673_1133_HM802934.1/1-445 GGTGGTGT-AGGATT-CACTCAATACGGAACCGCTGCTTACACTGACAACATTCTCGATG
Methanobacterium_thermaggregans_1_440_AY289750.1/1-440 GGTGGTGT-AGGATT-CACCCAGTACGCAACAGCAGCCTACACAGACAACATCCTCGACG
Methanothermobacter_wolfeii_259_719_AB300780.1/1-445 GGTGGTGT-TGGATT-CACCCAGTACGCAACAGCAGCCTACACAGATAACATTCTGGATG
Methanothermobacter_tenebrarum_259_723_AB523786.1/1-449 GGTGGTGT-AGGATT-CACACAATATGCAACAGCAGCATACACAGACAACATCCTAGACG
Methanothermobacter_crinale_18_503_HQ283274.1/1-470 GGTGGTGT-AGGATT-CACACAATATGCTACAGCAGCATACACAGACAACATCCTAGACG
Methanobacterium_aggregans_690_1116_KP006500.1/1-411 GGTGGTGT-TGGATT-CACACAGTACGCAACAGCTGCTTACACTGACAACATCCTTGACG
Methanobacterium_aarhusense_650_1124_AY386125.1/1-459 GGTGGTGT-AGGATT-CACACAATATGCTACCGCAGCATACACCGACAACATTCTTGACG
Methanobacterium_bryantii_259_719_AF313806.1/1-445 GGTGGTGT-AGGATT-CACTCAGTACGCTACAGCAGCATACACTGATAATATTCTTGATG
Methanococcus_voltae_5430_5930_X07793.1/1-485 GGTGGTGT-AGGATT-CACACAATATGCTACAGCATCATACACAGATGACATCTTAGATG
Methanothermococcus_thermolithot_1_414_AF414048.1/1-414 --------------------------------------TACACCGACGACATCTTAGACG
Methanococcus_maripaludis_684_1144_AB703637.1/1-445 GGTGGTGT-AGGATT-CACTCAATACGCTACCGCATCATACACCGACGATATCTTAGATG
Methanococcus_vannielii_4514_5014_M16893.1/1-485 GGTGGCGT-AGGTTT-CACACAATACGCTACAGCAGCATACACCGACGATATCTTGGACG
Methanococcus_aeolicus_1_464_AY354034.1/1-464 GGTGGTGT-CGGATT-CACACAGTATGCAACAGCAGCATACACTGATGACATCTTAGATG
Methanosphaera_sp._1_464_FJ982887.1/1-464 GGTGGTGT-CGGATT-CACACAATACGCAAGCGTAGCATACACAGATGAAATCTTAGACG
Methanosphaera_stadtmanae_1_414_AF414047.1/1-414 --------------------------------------TACACAGATGAAATATTAGATG

Methanopyrus_sp._18_510_KC618341.1/1-477 ACTACGTCTACCATGCTATCGACCTCATCAAAGATGAGT---AC------------GG--
Methanomassiliicoccus_luminyensis_1_456_strain_B10_HQ896500.1/1-455 ACTACACCTACTGGGCTCTTGACCTGATCAAGACCAAGT---AC------------GG--
Methanopyrus_kandleri_1_414_AF414042.1/1-414 ACTACGTGTACTACGGTCTCGAGTACGTCGAGGACAAGT---AC------------GG--
Methanosarcina_mazei_MH004454.1_RCompl_1_433/1-433 ACAACACCTTCTATGACGTTGACTACATCAACGACAAGT---ACAACGGTGCTGCAAA--
Methanosarcina_horonobensis_256_737_AB288266.1/1-466 ACAACGTGTACTACGACGTTGACTACATCAACGACAAGT---ACAACGGTGCTGCAAA--
Methanosarcina_subterranea_259_740_AB288268.1/1-466 ACAACGTGTACTATGACGTAGACTACATCAACGACAAGT---ACAACGGTGCTGCAAA--
Methanolobus_oregonensis_1_490_U22242.1/1-474 ACAACCTTTACTACAACATTGACTATATCAACGACAAGT---ACAATGATGCAGCAAA--
Methanolobus_zinderi_256_745_EU715818.1_RCompl/1-474 ACAACCTCTACTACAACGTTGACTACATCAACGACAAGT---ACGATGGTGCAGCAAA--
Methanolobus_taylorii_1_490_U22243.1/1-474 ACAACCTCTACTACAACGTTGACTACATCAACGACAAGT---ATGATGGTGCAGCAAA--
Methanolobus_profundi_687_1168_AB703629.1/1-466 ACAACCTCTACTACAACGTAGACTACATCAACGACAAGT---ACGATGGTGCAGCAAC--
Methanolobus_vulcani_1_490_U22245.1/1-474 ACAACCTCTACTACAACGTTGACTACATCAACGACAAGT---ACGATGGTGCAGCAAA--
Methanocella_conradii_1_465_JN081865.1/1-450 ACTTCACCTACTACGGCTATGACTATGCCAAGGGCAAGT---AC------------AA--
Methanocella_sp._279_692_KJ441441.1/1-398 ACTTCACATACTATGGCTACGACTACGCCAAGGGCAAGT---AC------------AA--
Methanocella_paludicola_240_681_AB300467.1/1-426 ACTTCACCTACTACGGTTATGACTACGCCAAGGGCAAGT---AC------------AA--
Methanomicrobium_mobile_1_438_AF414044.1/1-438 AGTTCACCTACTACGGTATGGACTACATTAAAGACAAAT---AC------------GG--
Methanoculleus_marisnigri_RCompl_1_436_MH004450.1/1-436 AGTACACCTACTATGGTATGGACTACATCAAGGACAAGT---AC------------AA--
Methanoculleus_palmolei_259_743_AB300784.1/1-469 AATTCACCTACTACGGTATGGACTACATCAAGGACAAGT---AC------------AA--
Methanoculleus_bourgensis_259_743_AB300787.1/1-469 AGTTCACCTACTACGGTATGGACTACATCAAGGACAAGT---AC------------AA--
Methanoculleus_chikugoensis_259_743_AB300779.1/1-469 AGTTCACCTACTACGGTATGGACTACATCAAGGACAAGT---AC------------AA--
Methanogenium_organophilum_1_501_DQ229160.1/1-487 A-TTCACCTACTACGGTATGGACTACCTGAAAGACAAGT---AC------------GA--
Methanogenium_boonei_15_520_DQ229161.1/1-490 AGTACACCTACTACGGTATGGACTACCTGAAGGACAAGT---AC------------GG--
Methanogenium_cariaci_1_433_DQ229157.1/1-420 AGTACACCTACTACGGTATGGACTACCTGAAGGACAAGT---AC------------GG--
Methanobacterium_sp._1_452_JQ917190.1/1-452 ACTTTGTATACTATGGTATGGAATACGTGGACGACAAAT---AC------------GG--
Methanobrevibacter_smithii_1_423_GU385700.1/1-423 ACTTCACTTACTATGGTAAAGATTACGTAGAAGACAAAT---AT------------GG--
Methanobrevibacter_oralis_1_414_LK054628.1/1-414 ACTTCACTTACTTCGGTAAAGATTATGTTGAAGATAAAT---AC------------GG--
Methanobrevibacter_ruminantium/1-470 ACTTCTCTTACTTCGGTAAAGATTACGTAGAAGACAAAT---AC------------GG--
Methanobrevibacter_sp._D5_mcrA_plasmid_365_874_KF214818.1/1-488 ACTTCACCTACTACGGTAAAGACTACGTCGAAGACAAAT---AC------------GG--
Methanobrevibacter_gottschalkii_1_471_EU919431.1/1-471 ACTTCACCTACTATGGTAAAGATTATGTCGAAGACAAAT---AT------------GG--
Methanobrevibacter_boviskoreani_1_424_KC865050.1/1-424 ACTTTACTTACTATGGTAAAGATTACGTAGAGGATAAAT---AT------------GG--
Methanobrevibacter_woesei_1_467_EU919432.1/1-467 ACTTTGCTTACTATGGTAAAGATTATGTAGAAGATAAAT---AT------------GG--
Methanobrevibacter_wolinii_1_395_KC865051.1/1-395 ---------------GTAAAGATTATGTAGAAGATAAAT---AT------------GG--
Methanobrevibacter_arboriphilus_1_414_AF414035.1/1-414 ATTTCTTATATTATGGTAAAGAATATGTCGAAGACAAAT---TT------------GG--
Methanobacterium_movens_673_1133_HM802934.1/1-445 ACTTTACCTACTATGGTAAAGAATACGTAGAAGACAAAT---TT------------GG--
Methanobacterium_thermaggregans_1_440_AY289750.1/1-440 ACTTCACCTACTTCGGTAAGGAGTATGTGGAAGACAAGT---AC------------GG--
Methanothermobacter_wolfeii_259_719_AB300780.1/1-445 ACTTCACCCACTTCGGTAAGGAGTACGTGGAAGACAAAT---AT------------GG--
Methanothermobacter_tenebrarum_259_723_AB523786.1/1-449 ACTTCACCTACTATGGTAGAGAATACGTCGAAGACAAGT---AT------------GGTG
Methanothermobacter_crinale_18_503_HQ283274.1/1-470 ACTTCACCTACTATGGTAGAGAATACGTCGAAGACAAGT---AT------------GG--
Methanobacterium_aggregans_690_1116_KP006500.1/1-411 ACTTCTGTTACTACGGTAAAGAGTACGTCGAAGACAAATTCGGT------------GG--
Methanobacterium_aarhusense_650_1124_AY386125.1/1-459 ACTTCACCTACTATGGTAAAGAATACGTCGAAGACAAAT---TC------------GG--
Methanobacterium_bryantii_259_719_AF313806.1/1-445 ACTTCACATATTTCGGTAGAGAATATGTAGAAGACAAAT---AT------------GG--
Methanococcus_voltae_5430_5930_X07793.1/1-485 ACTTCTCATACTACGGATACGAATACGTAGAGAAAAAAT---AC------------GG--
Methanothermococcus_thermolithot_1_414_AF414048.1/1-414 ACTTCGCATACTACGGATACGAATACGTAGAGAAGAAAT---AC------------GG--
Methanococcus_maripaludis_684_1144_AB703637.1/1-445 ACTTCTCATACTACGCACTCGACTACGTTGAGAAAAAAT---AC------------GG--
Methanococcus_vannielii_4514_5014_M16893.1/1-485 ATTTCTCATACTATGCACTTGACTACGTAGAGAAAAAAT---AC------------GG--
Methanococcus_aeolicus_1_464_AY354034.1/1-464 ATTTCTGCTACTATGGATTAGACTATGTAGAGAAAAAAT---TC------------GG--
Methanosphaera_sp._1_464_FJ982887.1/1-464 ACTTCATATACTACGGTAAAGACTACGTAGACAACAAAT---AC------------GG--
Methanosphaera_stadtmanae_1_414_AF414047.1/1-414 ACTTCATTTACTATGGTAAAGACTACGTAGAAGGTAAAT---AT------------GG--

Methanopyrus_sp._18_510_KC618341.1/1-477 -------CGGCTTCTGC-GGTGT-------------------------------------
Methanomassiliicoccus_luminyensis_1_456_strain_B10_HQ896500.1/1-455 -------CGGGCTCTGC-AAGAG-------------------------------------
Methanopyrus_kandleri_1_414_AF414042.1/1-414 ----------AATCGCC-GAGGC-------------------------------------
Methanosarcina_mazei_MH004454.1_RCompl_1_433/1-433 ----------CCTCGGAACTGAC-------------------------------------
Methanosarcina_horonobensis_256_737_AB288266.1/1-466 ----------CGTAGGCAAGGAC-------------------------------------
Methanosarcina_subterranea_259_740_AB288268.1/1-466 ----------CATCGGCAAGGAC-------------------------------------
```

```
Methanolobus_oregonensis_1_490_U22242.1/1-474                        ----------GACCGGTACAGAT-------------------------------------
Methanolobus_zinderi_256_745_EU715818.1_RCompl/1-474                 ----------CAAGGGTAAAGAC-------------------------------------
Methanolobus_taylorii_1_490_U22243.1/1-474                           ----------CAAGGGTACAGAC-------------------------------------
Methanolobus_profundi_687_1168_AB703629.1/1-466                      ----------AACAGGTACAGAC-------------------------------------
Methanolobus_vulcani_1_490_U22245.1/1-474                            ----------CAAGGGTACAGAC-------------------------------------
Methanocella_conradii_1_465_JN081865.1/1-450                         ----------GAT--GC-C-GAC-------------------------------------
Methanocella_sp._279_692_KJ441441.1/1-398                            ----------GAT--GC-C-GAC-------------------------------------
Methanocella_paludicola_240_681_AB300467.1/1-426                     ----------GATCGGC-CAGAC-------------------------------------
Methanomicrobium_mobile_1_438_AF414044.1/1-438                       ----------TGTTGAC-TACAA-----------GAACCCATCCCCCGCAAAGCTCG---
Methanoculleus_marisnigri_RCompl_1_436_MH004450.1/1-436              ----------GGTCGAC-TGGGC-----------AAACCC----------GAACGAGAAG
Methanoculleus_palmolei_259_743_AB300784.1/1-469                     ----------GGTCGAC-TGGAA-----------GAACCC----------GAGCCCGAAG
Methanoculleus_bourgensis_259_743_AB300787.1/1-469                   ----------GGTCGAC-TGGAA-----------GAACCC----------GAGCGCGAAG
Methanoculleus_chikugoensis_259_743_AB300779.1/1-469                 ----------GGTCGAC-TGGAA-----------GAACCC----------GAGCCCCAAG
Methanogenium_organophilum_1_501_DQ229160.1/1-487                    ----------CTTCGAC-TACTCTACTCCATCTGCAAC-----------AGCAACCC---
Methanogenium_boonei_15_520_DQ229161.1/1-490                         ----------CTTCGAC-TACTC-----------CAACCCGGACCCCGCAGCAACCA---
Methanogenium_cariaci_1_433_DQ229157.1/1-420                         ----------CTTCGAC-TACTC-----------C-ACCCGGACCCCGCAGCAACCA---
Methanobacterium_sp._1_452_JQ917190.1/1-452                          ----------TATCTGC-GGAAC-------------------------------------
Methanobrevibacter_smithii_1_423_GU385700.1/1-423                    -TGG------ATTAACT-GAAGC-------------------------------------
Methanobrevibacter_oralis_1_414_LK054628.1/1-414                     -TGG------ATTAACT-CAAGC-------------------------------------
Methanobrevibacter_ruminantium/1-470                                 -AGA------CTTATGT-TCCGC-------------------------------------
Methanobrevibacter_sp._D5_mcrA_plasmid_365_874_KF214818.1/1-488      -AGA------ATTATGC-GCAGC-------------------------------------
Methanobrevibacter_gottschalkii_1_471_EU919431.1/1-471               -TGA------TTTATGT-GCTGC-------------------------------------
Methanobrevibacter_boviskoreani_1_424_KC865050.1/1-424               -CGG------ATTAACC-GAAGC-------------------------------------
Methanobrevibacter_woesei_1_467_EU919432.1/1-467                     -T---------TTAACC-AAAGC-------------------------------------
Methanobrevibacter_wolinii_1_395_KC865051.1/1-395                    -T---------TTAACC-AAAGC-------------------------------------
Methanobrevibacter_arboriphilus_1_414_AF414035.1/1-414               ----------AATATGT-GAAGC-------------------------------------
Methanobacterium_movens_673_1133_HM802934.1/1-445                    ----------AATGTGT-GAAGC-------------------------------------
Methanobacterium_thermaggregans_1_440_AY289750.1/1-440               ----------ACTCTGT-GAGGC-------------------------------------
Methanothermobacter_wolfeii_259_719_AB300780.1/1-445                 ----------ACTCTGT-GAGGC-------------------------------------
Methanothermobacter_tenebrarum_259_723_AB523786.1/1-449              G---------ACTTGCA-GAAGC-------------------------------------
Methanothermobacter_crinale_18_503_HQ283274.1/1-470                  ----------ACTTGCA-GAAGC-------------------------------------
Methanobacterium_aggregans_690_1116_KP006500.1/1-411                 ----------ACTATGT-GAAGC-------------------------------------
Methanobacterium_aarhusense_650_1124_AY386125.1/1-459                ----------ATTATGT-GAAGC-------------------------------------
Methanobacterium_bryantii_259_719_AF313806.1/1-445                   ----------ACTTACC-GAAGC-------------------------------------
Methanococcus_voltae_5430_5930_X07793.1/1-485                        ----------AAGATGC-GGAAC-------------------------------------
Methanothermococcus_thermolithot_1_414_AF414048.1/1-414              ----------AATAAAC-AGTAC-------------------------------------
Methanococcus_maripaludis_684_1144_AB703637.1/1-445                  ----------TAGAATG-GGAAC-------------------------------------
Methanococcus_vannielii_4514_5014_M16893.1/1-485                     ----------TAGAATG-GGAAC-------------------------------------
Methanococcus_aeolicus_1_464_AY354034.1/1-464                        ----------TAGATGT-GGAAC-------------------------------------
Methanosphaera_sp._1_464_FJ982887.1/1-464                            ----------ATTATGT-CAAGC-------------------------------------
Methanosphaera_stadtmanae_1_414_AF414047.1/1-414                     ----TGG---ATTATGT-CAAGC-------------------------------------

Methanopyrus_sp._18_510_KC618341.1/1-477                             --------AA--------AACCCGATGACATGAA---------GAAGATCATGAAACTCG
Methanomassiliicoccus_luminyensis_1_456_strain_B10_HQ896500.1/1-455  --------CA--------AGCCCT---CCATGGA---------CCTGATGGAGAAGCTCG
Methanopyrus_kandleri_1_414_AF414042.1/1-414                         --------CG--------AGCCGA---GCATGGA---------CGTGGTGAAGGACGTCG
Methanosarcina_mazei_MH004454.1_RCompl_1_433/1-433                   --------AACAAGGTAAAGGCAA---CCCTCGA---------TGTAGTAAAAGACATCG
Methanosarcina_horonobensis_256_737_AB288266.1/1-466                 --------AACAAGATAAAGGCAA---CCCTCGA---------AGTCGTAAAGGACATCG
Methanosarcina_subterranea_259_740_AB288268.1/1-466                  --------AACAAGATAAAGGCAA---CTCTCGA---------AGTCGTAAAGGACATCG
Methanolobus_oregonensis_1_490_U22242.1/1-474                        --------AACAAGGTACAGGCAA---CACTTGA---------TGTTGTAAAGGATATCT
Methanolobus_zinderi_256_745_EU715818.1_RCompl/1-474                 --------AACAAGATCGCAGCAA---ACCTCGA---------TGTAGTAAAGGACATCG
Methanolobus_taylorii_1_490_U22243.1/1-474                           --------AACAAGGTAAAGGCAA---CCCTTGA---------CGTTGTAAAGGACATCG
Methanolobus_profundi_687_1168_AB703629.1/1-466                      --------AACAAGATAAAGGCAG---ACCTCGA---------AGTAGTAAAGGACATCG
Methanolobus_vulcani_1_490_U22245.1/1-474                            --------AACAAGGTAAAGGCAA---GCCTCGA---------TGTAGTAAAGGACATTG
Methanocella_conradii_1_465_JN081865.1/1-450                         --------CA--------AGCCCA---CTATGGA---------CATAGTCAACGACCTGG
Methanocella_sp._279_692_KJ441441.1/1-398                            --------CA--------AGGCTG---ACATGGA---------CGTAGTCAACGACCTGG
Methanocella_paludicola_240_681_AB300467.1/1-426                     --------CA--------AGCCGA---CCATGGA---------CATAGTCAACGACCTGT
Methanomicrobium_mobile_1_438_AF414044.1/1-438                       -------TAA--------AACCAA---CCCAGGA---------AGTTGTAAACGACATCG
Methanoculleus_marisnigri_RCompl_1_436_MH004450.1/1-436              GACAAGGTCA--------AGCCAA---CCCAGGA---------CATTGTCAACGACATCG
Methanoculleus_palmolei_259_743_AB300784.1/1-469                     GACAAGGTCA--------AGCCGA---CCCAGGA---------GATCGTCAACGACATGG
Methanoculleus_bourgensis_259_743_AB300787.1/1-469                   GACAAGATCA--------AGCCGA---CCCAGGA---------TGTCGTCAACGACATGG
Methanoculleus_chikugoensis_259_743_AB300779.1/1-469                 GACAAGATCA--------AGCCGA---CCCAGGA---------TATCGTCAACGACATGG
Methanogenium_organophilum_1_501_DQ229160.1/1-487                    -------TCG--------AGCCTA---CCCAGGA---------CCTCGTCAACGACCTTA
Methanogenium_boonei_15_520_DQ229161.1/1-490                         -------TCA--------CTCCTA---CCCAGGA---------GATCGTCAACGACCTTG
Methanogenium_cariaci_1_433_DQ229157.1/1-420                         -------TCA--------CTCCAA---CTCAGGA---------TGTCGTCAACGACCTTG
Methanobacterium_sp._1_452_JQ917190.1/1-452                          --------CA--------AAGCCA---GCACTGA---------AGTAGTTCACGACATAA
Methanobrevibacter_smithii_1_423_GU385700.1/1-423                    --------AC--------CTAATA---ACATGGACACTGTTCTTGATGTA---------G
Methanobrevibacter_oralis_1_414_LK054628.1/1-414                     --------AC--------CAAATA---ATATGGATACTGTTTTAGATGTT---------G
Methanobrevibacter_ruminantium/1-470                                 --------AC--------CTAACG---ACATGGACACCGTTCTTGACGTA---------G
Methanobrevibacter_sp._D5_mcrA_plasmid_365_874_KF214818.1/1-488      --------AC--------CTAACA---ACATGGACACCGTTCTTGATGTA---------G
Methanobrevibacter_gottschalkii_1_471_EU919431.1/1-471               --------AC--------CTAATA---ACATGGACACTGTTCTTGATGTA---------G
Methanobrevibacter_boviskoreani_1_424_KC865050.1/1-424               --------TC--------CTAACA---ACATGGATACTGTTCTTGATGTA---------G
Methanobrevibacter_woesei_1_467_EU919432.1/1-467                     --------AC--------CTAACA---ACATGGACACTGTTCTTGATGTT---------G
Methanobrevibacter_wolinii_1_395_KC865051.1/1-395                    --------AC--------CTAACA---ACATGGACACTGTTCTTGATGTT---------G
Methanobrevibacter_arboriphilus_1_414_AF414035.1/1-414               --------TC--------CAAACA---ACATGGACACTGTTCTTGATGTA---------G
Methanobacterium_movens_673_1133_HM802934.1/1-445                    --------AC--------CAAATA---CCATGGAAACCGTTCTGGATGTA---------G
Methanobacterium_thermaggregans_1_440_AY289750.1/1-440               --------AC--------CAAACA---CCATGGACACAGTCCTGGACGTT---------G
Methanothermobacter_wolfeii_259_719_AB300780.1/1-445                 --------AC--------CGAACA---CCATGGACACGGTCCTTGATATT---------G
Methanothermobacter_tenebrarum_259_723_AB523786.1/1-449              --------AC--------CTAACA---CCATGGACACAGTCCTAGACGTG---------G
Methanothermobacter_crinale_18_503_HQ283274.1/1-470                  --------GC--------CTAACA---CCATGGACACAGTCCTAGACATT---------G
Methanobacterium_aggregans_690_1116_KP006500.1/1-411                 --------TC--------CAAACG---ACATGGACACAGTACTGGACGTA---------G
Methanobacterium_aarhusense_650_1124_AY386125.1/1-459                --------AC--------CTAACA---ACATGGACACCGTGTTAGATGTG---------G
Methanobacterium_bryantii_259_719_AF313806.1/1-445                   --------GC--------CAAATA---CTATGGAAACAGTCCTTGATGTA---------G
Methanococcus_voltae_5430_5930_X07793.1/1-485                        --------TA--------AAGCTA---CAATGGA---------TGTAGTAGAAGACATCG
Methanothermococcus_thermolithot_1_414_AF414048.1/1-414              --------AA--------AACCAA---CAATGGA---------TGTTGTTGAAGACATTG
Methanococcus_maripaludis_684_1144_AB703637.1/1-445                  --------AA--------AAGCTA---CAATGGA---------TGTAGTAGAAGATGTTG
Methanococcus_vannielii_4514_5014_M16893.1/1-485                     --------TA--------AAGCTA---CAATGGA---------CGTAGTAGAAGACATTG
Methanococcus_aeolicus_1_464_AY354034.1/1-464                        --------AA--------AAGCTA---CAATGGA---------TGTTGTAGAAGATATTG
Methanosphaera_sp._1_464_FJ982887.1/1-464                            --------TG--------AAGCTA---ATTCCGA---------TGTTGTAAGAGACATCG
Methanosphaera_stadtmanae_1_414_AF414047.1/1-414                     --------TG--------AAGCAA---CTTCCGA---------AGTTGTAAAAGACATTG

Methanopyrus_sp._18_510_KC618341.1/1-477                             ACGATGAAGTCACTGCGTACGCACTTGAGATGTACGAGAGATACCCCGCAGTCATGGAGA
Methanomassiliicoccus_luminyensis_1_456_strain_B10_HQ896500.1/1-455  GCACCGAGATCAACTCCTACGCTCTGGAGATGTACGAGAGATACCCCGCCGCCATGGAAA
Methanopyrus_kandleri_1_414_AF414042.1/1-414                         CGACCGAGGTCACCCTGTACGGTCTGGAGCAGTACGAGCGGTACCCGGCCGCCATGGAGA
Methanosarcina_mazei_MH004454.1_RCompl_1_433/1-433                   CAACCGAGTCCACACTCTATGGTATCGAGACCTACGAGAAATTCCCAACAGCCCTTGAAG
Methanosarcina_horonobensis_256_737_AB288266.1/1-466                 CAACCGAATCCACACTCTACGGTATCGAGACCTACGAGAAATTCCCGACTGCCCTTGAAG
Methanosarcina_subterranea_259_740_AB288268.1/1-466                  CAACTGAGTCCACACTCTACGGTATCGAGACATACGAGAAGTTCCCGACTGCCCTTGAAG
Methanolobus_oregonensis_1_490_U22242.1/1-474                        CAACAGAGTCCACAATATATGGTCTTGAGAATTACGAGAAATACCCAACAACCCTTGAGG
Methanolobus_zinderi_256_745_EU715818.1_RCompl/1-474                 CAACAGAGTCCACAATCTACGGTCTCGAGAACTACGAGAAGTACCCAACAACCCTCGAAG
Methanolobus_taylorii_1_490_U22243.1/1-474                           CAACAGAATCCACTGTATACGGTCTTGAGAACTACGAGAAGTACCCAACAACCCTCGAAG
Methanolobus_profundi_687_1168_AB703629.1/1-466                      CAACAGAGTCCACGATCTACGGTCTCGAGAACTACGAGAAGTACCCAACAACCCTCGAAG
Methanolobus_vulcani_1_490_U22245.1/1-474                            CAACAGAGTCCACAATCTACGGTCTTGAGAACTACGAGAAATACCCAACAACCCTCGAAG
Methanocella_conradii_1_465_JN081865.1/1-450                         GCACTGAGGTCACCCTGTACGGCATCGAGCAGTACGAGAAGTACCCGACCACCCTCGAGG
Methanocella_sp._279_692_KJ441441.1/1-398                            GCACTGAGGTCACCCTGTATGGTATAGAGCAGTACGAGAAGTACCCGACCACCCTCGAGG
Methanocella_paludicola_240_681_AB300467.1/1-426                     CGACGGAAGTCACCCTGTATGGTATCGAGCAGTACGAGAAGTACCCGACCACCCTCGAGG
```

```
Methanomicrobium_mobile_1_438_AF414044.1/1-438                        CAACCGAAGTCAACCTTAACGGTATGGAGCAGTACGAACAGTATCCGACAATGATGGAAG
Methanoculleus_marisnigri_RCompl_1_436_MH004450.1/1-436               CAACCGAGGTGTGCCTGAACGGTATGGAGCAGTACGAGCAGTTCCCGACCCTGATGGAGG
Methanoculleus_palmolei_259_743_AB300784.1/1-469                      CGACCGAGGTCACCCTCAACGCCATGGAGCAGTACGAGCAGTTCCCGACCATGATGGAGG
Methanoculleus_bourgensis_259_743_AB300787.1/1-469                    CAACCGAGGTCACCCTCAACGCCATGGAGCAGTACGAGCAGTTCCCGACCATGATGGAAG
Methanoculleus_chikugoensis_259_743_AB300779.1/1-469                  CAACCGAGGTCACCCTCAACGCCATGGAGCAGTACGAGCAGTACCCGACCATGATGGAGG
Methanogenium_organophilum_1_501_DQ229160.1/1-487                     CAACCGAGGTCAACCTCAATGCAATGGAGCAGTACGAGCAGTTCCCAACACTCATGGAAG
Methanogenium_boonei_15_520_DQ229161.1/1-490                          CAACCGAGGTCAACCTCAATGCAATGGAGCAGTACGAGCAGTACCCCACCATGATGGAAG
Methanogenium_cariaci_1_433_DQ229157.1/1-420                          CAACTGAGGTTAACCTCAATGCAATGGAACAGTACGAACAGTACCCAACCATGATGGAAG
Methanobacterium_sp._1_452_JQ917190.1/1-452                           CCTCAGAAGTAACCATGTACGGACTGGAACAGTACGA---ATACCCAGCTCTCCTGGAAG
Methanobrevibacter_smithii_1_423_GU385700.1/1-423                     GTTCTGAAGTTTCATTCTACGCATTAGAACAATACGAAGAATACCCAGCTTTACTTGAAA
Methanobrevibacter_oralis_1_414_LK054628.1/1-414                      GTACTGAAGTTGCATTCTATGCATTAGAACAATACGAAGAATACCCAGCATTACTCGAAA
Methanobrevibacter_ruminantium/1-470                                  GTTCTGCAGTAACATTCTACTCATTAGAACAATACGAAGAATACCCAGCTTTACTTGAAA
Methanobrevibacter_sp._D5_mcrA_plasmid_365_874_KF214818.1/1-488       GTACTGAAGTAGCATTCTACGCATTAGAACAATACGAAGAATACCCAGCTTTACTTGAAA
Methanobrevibacter_gottschalkii_1_471_EU919431.1/1-471                GTTCTGAAGTAGCATTCTATGCATTAGATCAATACGAAGAATACCCAGCTTTACTTGAAA
Methanobrevibacter_boviskoreani_1_424_KC865050.1/1-424                GTTCTGAAGTAACCTTCTATGCATTAGAACAATATGATGAATACCCAGCTTTACTTGAAA
Methanobrevibacter_woesei_1_467_EU919432.1/1-467                      GTTCTGAAGTTACCTTCTACGGATTAGAACAATATGAAAAATTCCCTGCATTACTTGAAA
Methanobrevibacter_wolinii_1_395_KC865051.1/1-395                     GTTCTGAAGTTACCTTCTACGGATTAGAACAATATGAAAAATTCCCTGCATTACTTGAAA
Methanobrevibacter_arboriphilus_1_414_AF414035.1/1-414                GTTCTGAAGTAACTTTCTATGGATTAGAACAATACGAAGAATATCCTGCATTACTTGAAA
Methanobacterium_movens_673_1133_HM802934.1/1-445                     GTTCAGAAGTTACCTTCTATGCCCTGGAACAGTTTGAAGAATACCCAGCACTATTAGAAA
Methanobacterium_thermaggregans_1_440_AY289750.1/1-440                CGAGTGAGGTCACATTCTATGGACTTGAACAGTACGAGGAATACCCTGCACTCCTCGAGG
Methanothermobacter_wolfeii_259_719_AB300780.1/1-445                  CAACTGAGGTCACGTTCTACGGACTTGAACAGTACGAAGAATACCCAGCACTCCTGGAGG
Methanothermobacter_tenebrarum_259_723_AB523786.1/1-449               CAAGTGAAGTCACATTCTATTCCCTAGAACAGTATGAGGATTACCCATCCCTACTCGAGA
Methanothermobacter_crinale_18_503_HQ283274.1/1-470                   CAAGTGAAGTCACATTCTACAGCCTAGAACTGTATGAAGACTACCCATCATTACTTGAGA
Methanobacterium_aggregans_690_1116_KP006500.1/1-411                  GTTCTGAAGTAACCTTCTACTCACTTGAACAGTACGAAGAATACCCAGCACTTCTCGAGA
Methanobacterium_aarhusense_650_1124_AY386125.1/1-459                 CATCAGAAGTAACATTCTATGGCCTAGAACAATACGAAGAATACCCAGCACTCTTAGAAA
Methanobacterium_bryantii_259_719_AF313806.1/1-445                    CTTCAGAAGTTACATTCTATGGATTGGAACAGTATGAAGAATATCCAGCACTTCTTGAAG
Methanococcus_voltae_5430_5930_X07793.1/1-485                         CTTCAGAAGTTACACTCTACGCATTAGAACAGTATGATGAATACCCAGCATTATTAGAAG
Methanothermococcus_thermolithot_1_414_AF414048.1/1-414               CTACAGAAGTTACACTCTACTCATTAGAACAGTACGACGAATTCCCAACATTATTAGAAG
Methanococcus_maripaludis_684_1144_AB703637.1/1-445                   CTGGAGAAGTTACACTCTACGCATTAGAACAGTACGATGACTACCCAGCATTATTAGAAG
Methanococcus_vannielii_4514_5014_M16893.1/1-485                      CAAGTGAAGTTACACTCTACTCATTAGAACAGTACGATGAATACCCCGCATTATTAGAAG
Methanococcus_aeolicus_1_464_AY354034.1/1-464                         CAACAGAAGTTACACTTTATTCACTTGAACGATATGATGAATACCCAACATTATTGGAAG
Methanosphaera_sp._1_464_FJ982887.1/1-464                             CTAGTGAAGTAACATTATACGGATTAGAACAATACGA--AAT-CCCTGCAGCATTAGAAG
Methanosphaera_stadtmanae_1_414_AF414047.1/1-414                      CAAGTGAAGTAACCTTATACGGATTAGAACAATACGA--AAT-CCCTGCAGCATTAGAAG

Methanopyrus_sp._18_510_KC618341.1/1-477                              CCCACTTCGGTGGATCCCAGAGAGGTACCGTCACTGCAGCAGCAACCGGT---ATCGCAG
Methanomassiliicoccus_luminyensis_1_456_strain_B10_HQ896500.1/1-455 CCCACTTCGGTGGTTCCCAGCGTGCCACCGTCGCTGCCGCCGCCACCGGT---ATCGGCT
Methanopyrus_kandleri_1_414_AF414042.1/1-414                          CGCACTTCGGAGGTTCACAGCGAGCCGCCGTCTGTGCGGCCGCCGCGGGC---TGCAGTA
Methanosarcina_mazei_MH004454.1_RCompl_1_433/1-433                    ACCACTTCGGTGGGTCCCAGAGTGCAACCGTGCTTGCAGCTGCATCAGGC---GTTGCAT
Methanosarcina_horonobensis_256_737_AB288266.1/1-466                  ACCACTTCGGTGGATCCCAGAGAGCAACCGTGCTCGCAGCTGCAGCCGGT---GTCGCAT
Methanosarcina_subterranea_259_740_AB288268.1/1-466                   ACCACTTCGGTGGGTCCCAGAGAGCAACCGTGCTCGCAGCTGCAGCTGGT---GTTGCAT
Methanolobus_oregonensis_1_490_U22242.1/1-474                         ACCACTTCGGTGGATCACAGAGGGCAACAAGCCTTTCAGCAGCAGCTGGTTCCGCTGTAG
Methanolobus_zinderi_256_745_EU715818.1_RCompl/1-474                  ACCACTTCGGTGGATCACAGAGAGCAACCAGCCTTTCCGCAGCAGCAGGTTCCGCAGTAT
Methanolobus_taylorii_1_490_U22243.1/1-474                            ACCACTTTGGTGGATCACAGAGAGCAACAAGTCTTTCAGCAGCAGCTGGTTCCGCTGTAT
Methanolobus_profundi_687_1168_AB703629.1/1-466                       ACCACTTCGGTGGATCCCAGAGAGCAACAAGCCTTTCCGCAGCAGCTGGTTCAGCTGTAT
Methanolobus_vulcani_1_490_U22245.1/1-474                             ACCACTTCGGTGGATCACAGAGAGCAACAAGTCTTTCCGCAGCAGCAGGTTCCGCTGTAG
Methanocella_conradii_1_465_JN081865.1/1-450                          ACCACTTCGGCGGCTCTCAGAGGGCTACCGTCCTGGCAGCAGCGTCGGGC---GTCACTA
Methanocella_sp._279_692_KJ441441.1/1-398                             ACCACTTCGGTGGCTCGCAGAGGGCGACCGTGCTGGCAGCCGCATCGGGT---GTCACCA
Methanocella_paludicola_240_681_AB300467.1/1-426                      ACCACTTCGGTGGCTCGCAGAGGGCGACCGTGCTCTCGGCAGCAGCCGGT---GTTACCA
Methanomicrobium_mobile_1_438_AF414044.1/1-438                        ACCACTTCGGCGGTTCACAGCGTGCAAGTGTCCTTGCGGCATCCTGTGGT---ATCACCA
Methanoculleus_marisnigri_RCompl_1_436_MH004450.1/1-436               ACCACTTCGGCGGTTCCCAGCGTGCCGGTGTGCTCGCCGCCGCATGCGGT---CTGTCCA
Methanoculleus_palmolei_259_743_AB300784.1/1-469                      ACCACTTCGGCGGGTCCCAGCGCGCCGGCGTCATCGCCGCCGCATCCGGT---CTCACGA
Methanoculleus_bourgensis_259_743_AB300787.1/1-469                    ACCACTTCGGCGGGTCCCAGCGTGCTGGTGTCATCGCCGCTGCGTCCGGT---CTCACGA
Methanoculleus_chikugoensis_259_743_AB300779.1/1-469                  ACCACTTCGGCGGTTCCCAGCGTGCCGGTGTCATCGCCGCCGCGTCCGGT---CTGACGA
Methanogenium_organophilum_1_501_DQ229160.1/1-487                     ACCACTTCGGTGGTTCACAGCGTGCTGGTGTCATGGCTGCAGCCTGTGGT---CTTACCT
Methanogenium_boonei_15_520_DQ229161.1/1-490                          ACCACTTTGGCGGGTCACAGCGTGCTGGTGTTATGGCAGCAGCCTGTGGT---CTGACCT
Methanogenium_cariaci_1_433_DQ229157.1/1-420                          ACCACTTCGGCGGGTCACAGCGTGCTGGTGTTATGGCAGCAGCCTGTCGT---CTGACCT
Methanobacterium_sp._1_452_JQ917190.1/1-452                           ACCACTTTGGCGGTTCACAGCGAGCTGCAGTTGTTTCCGCTGCTGCCGGA---TGTTCCG
Methanobrevibacter_smithii_1_423_GU385700.1/1-423                     CTCACTTCGGTGGATCTCAAAGGGCTTCTGTTATTTCAGCGGCAGCTGGT---TGTTCCA
Methanobrevibacter_oralis_1_414_LK054628.1/1-414                      CTCACTTCGGTGGATCTCAAAGATCATCTGTTATTTCAGCAGCAGCTGGT---GCTTCAA
Methanobrevibacter_ruminantium/1-470                                  CTCACTTCGGTGGTTCTCAAAGAGCTGCTGTTGTATCTGCAGCTTCAGGT---ATTTCCA
Methanobrevibacter_sp._D5_mcrA_plasmid_365_874_KF214818.1/1-488       CTCACTTCGGTGGATCTCAAAGAGCTTCCGTTGTTTCCGCAGCTGCAGGT---TGTTCCA
Methanobrevibacter_gottschalkii_1_471_EU919431.1/1-471                CTCACTTCGGTGGATCTCAAAGAGCTTCAGTTATTTCTGCAGCTGCTGGT---TGTTCCA
Methanobrevibacter_boviskoreani_1_424_KC865050.1/1-424                CCCAATTCGGTGGATCTCAAAGAGCTGCTGTTACTGCAGCTGCTTCCGGT---TGTTGTA
Methanobrevibacter_woesei_1_467_EU919432.1/1-467                      CTCAATTCGGTGGATCCCAAAGGGCTGCTGTTGTTGCAGCTGCTGCAGGT---ATTTCAA
Methanobrevibacter_wolinii_1_395_KC865051.1/1-395                     CTCAATTCGGTGGATCCCAAAGGGCTGCTGTTGTTGCAGCTGCTGCAGGT---ATTTCAA
Methanobrevibacter_arboriphilus_1_414_AF414035.1/1-414                CCCAATTCGGTGGTTCTCAAAGAGCATCTGTTGTTTCAGCCGCTGCTGGT---TGTGCAA
Methanobacterium_movens_673_1133_HM802934.1/1-445                     CCCTCTTCGGTGGATCTCAAAGAGCTTCCATCGTAGCTGCAGCTGCTGGT---GCATCAA
Methanobacterium_thermaggregans_1_440_AY289750.1/1-440                ACCAGTTTGGTGGATCCCAGAGGGCAGCAGTTGTTGCAGCTGCAGCCGGA---TGTTCCA
Methanothermobacter_wolfeii_259_719_AB300780.1/1-445                  ACCAGTTCGGAGGATCCCAGAGGGCAGCCGTAGTTGCAGCCGCAGCTGGA---TGTTCAA
Methanothermobacter_tenebrarum_259_723_AB523786.1/1-449               CACAATTTGGAGGATCACAGAGGGCAGCTGTCGCAGCAGCAGCATCCGCT---TGTTCAA
Methanothermobacter_crinale_18_503_HQ283274.1/1-470                   CACAGTTCGGCGGATCACAAAGGGCAGCTGTCGCAGCAGCAGCAGCCGGT---TGTTCAA
Methanobacterium_aggregans_690_1116_KP006500.1/1-411                  CCCAGTTCGGTGGATCACAGAGGGCCGCAGTTGCAGCAGCTGCAGCAGGA---TGTTCCA
Methanobacterium_aarhusense_650_1124_AY386125.1/1-459                 CACAGTTCGGTGGATCACAAAGAGCAGCTGTAGCTGCTGCTGCTGCTGCA---TGTTCAA
Methanobacterium_bryantii_259_719_AF313806.1/1-445                    ATCAGTTCGGTGGATCACAGAGAGCAGCAGTTACTGCTGCAGCTGCAGGT---TGTTCAA
Methanococcus_voltae_5430_5930_X07793.1/1-485                         ACCACTTCGGTGGCTCACAAAGAGCTGCTGTTGCTGCTGCTGCAGCTGGT---ATTTCAG
Methanothermococcus_thermolithot_1_414_AF414048.1/1-414               ACCACTTCGGAGGTTCCCAAAGAGCTGCTGTTGCTGCAGCTGCTTCAGGT---ATCTCAG
Methanococcus_maripaludis_684_1144_AB703637.1/1-445                   ACCACTTCGGTGGATCACAAAGAGCTGCAGTTGCTGCTGCTGCATCAGGT---ATTGGTG
Methanococcus_vannielii_4514_5014_M16893.1/1-485                      ACCACTTTGGTGGATCACAGAGAGCTGCAGTTGCTGCTGCTGCATCCGGT---ATCGGTG
Methanococcus_aeolicus_1_464_AY354034.1/1-464                         ACCACTTTGGAGGTTCCCAAAGAGCAGCAGTTGCAGCAGCAGCGGCTGGT---GTTTCAG
Methanosphaera_sp._1_464_FJ982887.1/1-464                             ACCACTTCGGTGGATCACAGAGAGCAGCAGTTCTCGCAGCAGGTGCAGGT---TGTTCAG
Methanosphaera_stadtmanae_1_414_AF414047.1/1-414                      ACCACTTTGGTGGATCACAAAGAGCAGCAGTTCTTGCAGCAGGTGCAGGT---TGTTCAG

Methanopyrus_sp._18_510_KC618341.1/1-477                              CTGCTATGGCAACCGGAAACGCCGATATCGGTGTGAACGGATGGTATCTGTCCATGCTCC
Methanomassiliicoccus_luminyensis_1_456_strain_B10_HQ896500.1/1-455 GCGCGATGGCCACCGGCAACGCCGACTTCGGTGTCAACGGCTGGTATCTGTCCATGCTCC
Methanopyrus_kandleri_1_414_AF414042.1/1-414                          CCGCCTTCGCGACCGGTCACGCGCAGGCAGGACTCAACGGTTGGTACCTGTCGCAGATCC
Methanosarcina_mazei_MH004454.1_RCompl_1_433/1-433                    GCGCTCTTGCAACCGGAAACGCAAATGCTGGTCTCTCAGGCTGGTACCTCTCCATGTATG
Methanosarcina_horonobensis_256_737_AB288266.1/1-466                  GTGCACTCGGAACTGCAAACGCAAATGCCGGTCTCTCAGGCTGGTACCTCTCCATGTACC
Methanosarcina_subterranea_259_740_AB288268.1/1-466                   GCTCCCTCGGAACTGCAAACGCAAACGCTGGTCTGTCCGGCTGGTACCTCTCCATGTACC
Methanolobus_oregonensis_1_490_U22242.1/1-474                         ---CAATTGCAACAGGTAACGGAAACGCCGGTCTGTCCGGATGGTATCTCTCAATGTACC
Methanolobus_zinderi_256_745_EU715818.1_RCompl/1-474                  ---CAATTGCAACCGGAAACGGAAACGCCGGTCTGTCCGGATGGTACCTTTCAATGTACC
Methanolobus_taylorii_1_490_U22243.1/1-474                            ---CTCTGGCAACAGGAAACGGAAACGCTGGTCTGTCAGGATGGTACCTCTGTATGTACT
Methanolobus_profundi_687_1168_AB703629.1/1-466                       ---CCCTGGCAACAGGAAACGGAAACGCTGGTCTCTCCGGATGGTACCTCTGTATGTACC
Methanolobus_vulcani_1_490_U22245.1/1-474                             ---CTCTTGCAACAGGAAACGGAAACGCTGGTCTTTCCGGATGGTACCTCTGTATGTACC
Methanocella_conradii_1_465_JN081865.1/1-450                          CTGCGCTGGCCACGGGCAGCTCGAACGCTGGCCTGTCGGCCTGGTACCTGTCCATGTACC
Methanocella_sp._279_692_KJ441441.1/1-398                             CCGCTCTGGCGACTGGTAACTCGAACGCTGGCCTGTCGGCCTGGTACCTGTCCATGTACC
Methanocella_paludicola_240_681_AB300467.1/1-426                      CCGCTATCGCGACCGGTAACTCCAACGCTGGCCTGTCCGGCTGGTACATGTCAATGTACC
Methanomicrobium_mobile_1_438_AF414044.1/1-438                        CATCAATCGCAACAGGTAACTCCAACGCAGGTCTCAACGCATGGTACCTTTCGATGCTCA
Methanoculleus_marisnigri_RCompl_1_436_MH004450.1/1-436               CCTCGATCGCAACCGGAAACTCCAACGCCGGTCTGAACGCCTGGTACCTCTGCATGCTCC
Methanoculleus_palmolei_259_743_AB300784.1/1-469                      CCTCCATCGCAACCGGAAACTCGAATGCCGGTCTGAACGGCTGGTATCTCTCCATGCTCC
Methanoculleus_bourgensis_259_743_AB300787.1/1-469                    CCGCCATCGCAACCGGCAACTCCAACGCCGGTCTCAACGGATGGTATCTCTCGATGCTCC
Methanoculleus_chikugoensis_259_743_AB300779.1/1-469                  CCGCCATCGCGACCGGAAACTCGAACGCCGGCCTGAACGCCTGGTACCTCTCCATGCTCC
Methanogenium_organophilum_1_501_DQ229160.1/1-487                     GTTCCATCGGTACCGGAAACTCCAATGCCGGTCTGAACGGATGGTACCTTTCCATGCTTA
Methanogenium_boonei_15_520_DQ229161.1/1-490                          GTTCCATCGGTACCGGAAACTCCAATGCCGGTCTGAACGGATGGTACCTTTCCATGCTTA
Methanogenium_cariaci_1_433_DQ229157.1/1-420                          CTTCCATTGGTACCGGAAACTCCAATGCCGGTCTGAACGGATGGTATCTTTCC-------
```

```
Methanobacterium_sp._1_452_JQ917190.1/1-452                          TTGCTTTCGCAACTGGAAACTCCAACGCCGGAATCAACGGATGGTACTTAGCCA-GATCC
Methanobrevibacter_smithii_1_423_GU385700.1/1-423                    CTGCATTCGCTACTGGTAATGCTCAAACTGGTTTAAGTGCATGGTACTTAGGTATGTACT
Methanobrevibacter_oralis_1_414_LK054628.1/1-414                     CTGCATTTGCTACTGGTAATGCTCAAACTGGTTTAAGTGCATGGTACTTATCTATGTATT
Methanobrevibacter_ruminantium/1-470                                 CTGCATTCGCAACTGGAAACGCACAAACCGGTTTATCTGCATGGTACTTAGCACAATACT
Methanobrevibacter_sp._D5_mcrA_plasmid_365_874_KF214818.1/1-488      CTGCATTCGCTACTGGTAATGCACAAACTGGTTTAAGCGCATGGTACTTAGCTATGTACT
Methanobrevibacter_gottschalkii_1_471_EU919431.1/1-471               CTGCATTCGCTACTGGTAATGCACAAACTGGTTTAAGCGGATGGTACTTATCCATGTACT
Methanobrevibacter_boviskoreani_1_424_KC865050.1/1-424               CTGCATTTGCAACTGGTAACTCTCAAACTGGTTTAAGTGCATGGTACTTATCACAATACT
Methanobrevibacter_woesei_1_467_EU919432.1/1-467                     CTGCATTTGCTACTGGAAATGCTCAAACTGGTTTAAGTGCATGGTACTTATCTCAATACT
Methanobrevibacter_wolinii_1_395_KC865051.1/1-395                    CTGCATTTGCTACTGGAAATGCTCAAACTGGTTTAAGTGCATGGTACTTATCTCAATACT
Methanobrevibacter_arboriphilus_1_414_AF414035.1/1-414               CAGCTTTCGCTACTGGAAATTCCCAAACTGGTTTAAGCGCATGGTATTTATCTATGTACT
Methanobacterium_movens_673_1133_HM802934.1/1-445                    CTGCATTTGCTACTGCTAATGCTCAAACTGGTCTAAGTGCATGGTACCTATCTATGTACC
Methanobacterium_thermaggregans_1_440_AY289750.1/1-440               CAGCCTTCGCAACAGCAAATGCCCAGACAGGTCTCAGTGGATGGTACCTCTCAATGTACC
Methanothermobacter_wolfeii_259_719_AB300780.1/1-445                 CAGCATTTGCAACAGGAAACGCCCAGACAGGTCTGAGTGGATGGTACCTTTCAATGTACC
Methanothermobacter_tenebrarum_259_723_AB523786.1/1-449              CAGGATTCGCAACAGGAAACTCACAGACCGCATTAAGCGGATGGTACTTGTCAATGTACT
Methanothermobacter_crinale_18_503_HQ283274.1/1-470                  CCGGATTCGCAACAGGAAACTCACAGACCGCACTAAGCGGATGGTACCTATCAATGTACC
Methanobacterium_aggregans_690_1116_KP006500.1/1-411                 CAGGATTCGCAACTGGTAACGCTCAGACAGGTTTAAGCGCATGGTACTTATCCATGTACT
Methanobacterium_aarhusense_650_1124_AY386125.1/1-459                CTGGATTTGCTACTGGTAACGCACAAACTGCGTTAAGCGGTTGGTATTTATCCATGTACT
Methanobacterium_bryantii_259_719_AF313806.1/1-445                   CTGCATTTGCAACTGGAAATGCACAAACTGGTTTAAGTGGATGGTACTTATCCATGTACT
Methanococcus_voltae_5430_5930_X07793.1/1-485                        TATGTATGGCAACCGGTAACTCAAACGCTGGTGTTAACGGCTGGTACTTATCACAAATAT
Methanothermococcus_thermolithot_1_414_AF414048.1/1-414              TATGTATGGCTACAGGAAACTCAAACGCTGGTGTTAACGGCTGGTACTTAAGCCAAATCA
Methanococcus_maripaludis_684_1144_AB703637.1/1-445                  TATGTATGGCAACAGGAAACTCAAACGCTGGTGTAAACGGCTGGTACTTATCACAAATCT
Methanococcus_vannielii_4514_5014_M16893.1/1-485                     TATGTATGGCAACAGGTAACTCAAACGCTGGTGTAAACGGCTGGTACTTATCACAAATAT
Methanococcus_aeolicus_1_464_AY354034.1/1-464                        TATGTATGGCAACAGGAAACTCAAATGCAGGAGTTAATGGATGGTATTTAGC-CAAATAA
Methanosphaera_sp._1_464_FJ982887.1/1-464                            TAGCATTCGCAACTGCTAACTCTAACGCAGGTGTAAACGGATGGTACTTAAGTCAATTAT
Methanosphaera_stadtmanae_1_414_AF414047.1/1-414                     TAGCATTCGCAACTGCTAACTCTAACGCAGGTGTAAATGGATGGTACTTAAGTCAATTAT

Methanopyrus_sp._18_510_KC618341.1/1-477                             AGCACAAGGAGAGGACCGGAAGGCTCGGATTCTACGGATTCGATCTGCAGGATCAGTGCG
Methanomassiliicoccus_luminyensis_1_456_strain_B10_HQ896500.1/1-455 AGCACAAGGAGAGGCACGGACGCCTTGGCTTCTACGGCTACGACCTGCAGGACCAGTGCG
Methanopyrus_kandleri_1_414_AF414042.1/1-414                         TGCACAAGGAGGGTCACGGTCGTCTAGGATTCTACGGGTACGCCCTACAGGACCAGTGTG
Methanosarcina_mazei_MH004454.1_RCompl_1_433/1-433                   TCCACAAGGAAGCATGGGGCAGACTCGGATTCTTCGGTTTCGACCTGCAGGATCAGTGTG
Methanosarcina_horonobensis_256_737_AB288266.1/1-466                 TGCACAAGGAAGCATGGGGCAGACTCGGATTCTTCGGATACGACCTCCAGGACCAGTGCG
Methanosarcina_subterranea_259_740_AB288268.1/1-466                  TGCACAAGGAAGCATGGGGCCGTCTCGGCTTCTTCGGATACGACCTGCAGGACCAGTGCG
Methanolobus_oregonensis_1_490_U22242.1/1-474                        TGCACAAGGAAGCACACGGTCGTCTTGGCTTCTTCGGATACGACCTGCAGGACCAGTGTG
Methanolobus_zinderi_256_745_EU715818.1_RCompl/1-474                 TGCACAAGGAAGCACTCGGAAGACTCGGTTTCTTCGGATACGACCTGCAGGACCAGTGTG
Methanolobus_taylorii_1_490_U22243.1/1-474                           TGCACAAGGAAGCACTCGGAAGACTCGGTTTCTTCGGATACGACCTGCAGGACCAGTGTG
Methanolobus_profundi_687_1168_AB703629.1/1-466                      TCCACAAGGAAGCACACGGCCGTCTCGGTTTCTTCGGATACGACCTGCAGGACCAGTGTG
Methanolobus_vulcani_1_490_U22245.1/1-474                            TCCACAAGGAAGCACACGGCCGTCTCGGTTTCTTCGGATACGACCTGCAGGACCAGTGTG
Methanocella_conradii_1_465_JN081865.1/1-450                         TGCACAAGGAGGCATGGGGCAGGCTGGGCTTCTTCGGCTATGACCTGCAGGACCAGTGCG
Methanocella_sp._279_692_KJ441441.1/1-398                            TGCACAAGGAAGCATGGGGCAGGCTGGGCTTCTTCGGCTACGACCTGCAGGACCAGTGCG
Methanocella_paludicola_240_681_AB300467.1/1-426                     TGCACAAGGAAGCATGGGGCAGGCTGGGCTTCTTCGGCTACGACCTGCAGGACCAGTGCG
Methanomicrobium_mobile_1_438_AF414044.1/1-438                       TGCACAAGGGAGGCTGGTCACGTCTCGGATTCTTCGGATACGATCTTCAGGACCAGTGCG
Methanoculleus_marisnigri_RCompl_1_436_MH004450.1/1-436              TGCACAAGGAAGGCTGGTCACGTCTCGGATTCTTCGGATACGACCTGCAGGACCAGTGCG
Methanoculleus_palmolei_259_743_AB300784.1/1-469                     TGCACAAGGACGGATGGTCGCGTCTCGGATTCTTCGGCTACGACCTCCAGGACCAGTGCG
Methanoculleus_bourgensis_259_743_AB300787.1/1-469                   TGCACAAGGACGGCTGGTCGCGTCTCGGCTTCTTCGGCTACGACCTCCAGGACCAGTGCG
Methanoculleus_chikugoensis_259_743_AB300779.1/1-469                 TGCACAAGGACGGATGGTCGCGTCTCGGCTTCTTCGGCTACGACCTCCAGGACCAGTGCG
Methanogenium_organophilum_1_501_DQ229160.1/1-487                    TGCACAAGGAAGGATGGTCACGTCTTGGTTTCTTCGGATACGATCTTCAGGACCAGTGTG
Methanogenium_boonei_15_520_DQ229161.1/1-490                         TGCACAAGGAAGGCTGGTCACGTCTTGGTTTCTTCGGATACGATCTTCAGGACCAGTGTG
Methanogenium_cariaci_1_433_DQ229157.1/1-420                         ---------------------------------------------------------GTG
Methanobacterium_sp._1_452_JQ917190.1/1-452                          TACACAAAGAAGCCCACAGCCGACTTGGTTTCTACGGTTACGACCTGCAGGACCAGTGTG
Methanobrevibacter_smithii_1_423_GU385700.1/1-423                    TACACAAAGAACAACATTCCAGATTAGGTTTCTACGGATACGATTTACAAGATCAATGTG
Methanobrevibacter_oralis_1_414_LK054628.1/1-414                     TACACAAAGAACAACATTCCAGATTAGGTTTCTATGGTTTCGATTTACAAGATCAATGTG
Methanobrevibacter_ruminantium/1-470                                 TACACAAAGAACAACATTCCAGATTAGGATTCTACGGTTACGACTTGCAAGATCAATGTG
Methanobrevibacter_sp._D5_mcrA_plasmid_365_874_KF214818.1/1-488      TACACAAAGAACAACACTCCAGATTAGGATTCTACGGTTTCGATTTACAAGACCAATGTG
Methanobrevibacter_gottschalkii_1_471_EU919431.1/1-471               TACACAAAGAACAACATTCCAGATTAGGTTTCTACGGTTTCGATTTACAAGATCAATGTG
Methanobrevibacter_boviskoreani_1_424_KC865050.1/1-424               TACACAAAGAACAACACTCCAGATTAGGATTCTATGGTTACGATTTACAAGATCAATGTG
Methanobrevibacter_woesei_1_467_EU919432.1/1-467                     TACACAAAGAACAACATTCCAGATTAGGTTTCTATGGTTACGATTTACAAGATCAATGTG
Methanobrevibacter_wolinii_1_395_KC865051.1/1-395                    TACACAAAGAACAACATTCCAGATTAGGTTTCTATGGTTACGATTTACAAGATCAATGTG
Methanobrevibacter_arboriphilus_1_414_AF414035.1/1-414               TACACAAAGAACAACACTCTAGATTAGGATTCTATGGTTACGATTTACAAGATCAATGTG
Methanobacterium_movens_673_1133_HM802934.1/1-445                    TGCACAAAGAACAACACTCCAGACTTGGATTCTACGGATACGATCTGCAAGATCAGTGTG
Methanobacterium_thermaggregans_1_440_AY289750.1/1-440               TGCACAAGGAACAGCACTCAAGGCTCGGATTCTATGGTTACGACCTCCAGGACCAGTGTG
Methanothermobacter_wolfeii_259_719_AB300780.1/1-445                 TCCACAAGGAACAGCACTCCAGGCTTGGATTCTATGGTTACGACCTGCAGGACCAGTGCG
Methanothermobacter_tenebrarum_259_723_AB523786.1/1-449              TACACAAGGAGCAGCACGCAAGACTTGGATTCTACGGATACGACCTCCAAGACCAGTGTG
Methanothermobacter_crinale_18_503_HQ283274.1/1-470                  TACATAAAGAACAGCACGCAAGACTCGGATTTTATGGATACGACCTACAAGACCAGTGTG
Methanobacterium_aggregans_690_1116_KP006500.1/1-411                 TACACAAAGAACAGCACTCACGTCTTGGTTTCTACGGATACGACCTTCAGGATCAGTGTG
Methanobacterium_aarhusense_650_1124_AY386125.1/1-459                TGCACAAAGAACAACACTCCAGACTCGGTTTCTATGGATACGACCTTCAAGATCAGTGTG
Methanobacterium_bryantii_259_719_AF313806.1/1-445                   TACACAAAGAACAGCATTCCAGACTCGGATTCTACGGATACGACCTTCAGGATCAGTGTG
Methanococcus_voltae_5430_5930_X07793.1/1-485                        TACACAAAGAATACCACAGCAGACTTGGATTCTACGGTTACGACTTACAAGACCAATGTG
Methanothermococcus_thermolithot_1_414_AF414048.1/1-414              TGCACAAAGAATACCACAGCAGACTTGGATTCTACGGATACGACTTACAAGACCAGTGTG
Methanococcus_maripaludis_684_1144_AB703637.1/1-445                  TACACAAAGAATACCACAGCAGACTCGGATTCTACGGTTACGACTTGCAGGACCAGTGTG
Methanococcus_vannielii_4514_5014_M16893.1/1-485                     TACACAAAGAATACCACAGTAGACTTGGATTCTATGGTTACGACTTGCAGGACCAATGTG
Methanococcus_aeolicus_1_464_AY354034.1/1-464                        TGCATAAAGAATACCACAGTAGGTTAGGATTCTACGGATACGACTTACAAGACCAGTGTG
Methanosphaera_sp._1_464_FJ982887.1/1-464                            TACATAAAGAAGGACACAGCAGATTAGGATTCTACGGTTACGACTTACAAGATCAATGTG
Methanosphaera_stadtmanae_1_414_AF414047.1/1-414                     TACATAAAGAAGGTCACAGTAGATTAGGATTCTATGGTTACGACTTACAAGATCAATGTG

Methanopyrus_sp._18_510_KC618341.1/1-477                             GTTCTTCCAACTCGTTCTCCT-ACAGGTCCGATGAGG-----G-----TCT---------
Methanomassiliicoccus_luminyensis_1_456_strain_B10_HQ896500.1/1-455 GTTCCGCCAACTCGTTCGCCT-ACAGGAGCGACGAGG-----G-----CCT---------
Methanopyrus_kandleri_1_414_AF414042.1/1-414                         GTGCGGCCAACTCGCTGAGCG-TGAGGAGCGACGAGG-----G-----ACT---------
Methanosarcina_mazei_MH004454.1_RCompl_1_433/1-433                   GTGCCACAAACGTTCTGTCCT-ACCAGGGCGACGAAG-----G-----TCT---------
Methanosarcina_horonobensis_256_737_AB288266.1/1-466                 GTGCTACAAACGTTCTGTCCT-ACCAGGGCGACGAAG-----G-----TCT---------
Methanosarcina_subterranea_259_740_AB288268.1/1-466                  GTGCCACAAACGTTCTGTCCT-ACCAGGGCGACGAAG-----G-----TCT---------
Methanolobus_oregonensis_1_490_U22242.1/1-474                        GTGCCACAAACGTTCTCTCAT-ACCAGTCCGACGAAG-----G-----TCT---------
Methanolobus_zinderi_256_745_EU715818.1_RCompl/1-474                 GTGCTACAAACGTACTCTCCT-ACCAGTCCGACGAAG-----G-----TCT---------
Methanolobus_taylorii_1_490_U22243.1/1-474                           GTGCAACAAACGTTCTCTCAT-ACCAGTCCGACGAAG-----G-----TCT---------
Methanolobus_profundi_687_1168_AB703629.1/1-466                      GTGCTACAAACGTACTCTCAT-ACCAGTCCGACGAAG-----G-----TCT---------
Methanolobus_vulcani_1_490_U22245.1/1-474                            GTGCTACAAACGTACTCTCAT-ACCAGTCCGACGAAG-----G-----TCT---------
Methanocella_conradii_1_465_JN081865.1/1-450                         GTGCCACCAACGTGTTCTCCT-GCCGCTCCGACGAGG-----GCGCGATGT---------
Methanocella_sp._279_692_KJ441441.1/1-398                            GCGCGACCAACGTGTTCTC-----------------------------------------
Methanocella_paludicola_240_681_AB300467.1/1-426                     GTGCGACCAACGTGTTCTCGT-GCCGCTCTGATGAGG-----GCGCCATT----------
Methanomicrobium_mobile_1_438_AF414044.1/1-438                       GTTCGGCAAACTCACTCTCTA-TGGAGCCTGATCGCG-----G-----TCT---------
Methanoculleus_marisnigri_RCompl_1_436_MH004450.1/1-436              GTTCCACCAACTCGCTCTCTG-TCAGGCCTGACGAGG-----G-----TGC---------
Methanoculleus_palmolei_259_743_AB300784.1/1-469                     GCTCCGCAAACTCGCTCTCCA-TCCGTGGAGACGAGG-----G-----CGC---------
Methanoculleus_bourgensis_259_743_AB300787.1/1-469                   GGTCTGCAAACTCGCTCTCCA-T-------------G-----G-----AGCCCGACCGCG
Methanoculleus_chikugoensis_259_743_AB300779.1/1-469                 GGTCCGCGAACTCCCTCTCCA-T-------------G-----G-----AGTCCGACCGCG
Methanogenium_organophilum_1_501_DQ229160.1/1-487                    GTTCAGCAAACTCACTCNCTA-TGGACGCAGACCGCG-----G-----TCT---------
Methanogenium_boonei_15_520_DQ229161.1/1-490                         GTTCAGCAAACTC-CCTTTCATTGGAGCCTGACCGCG-----G-----TCT---------
Methanogenium_cariaci_1_433_DQ229157.1/1-420                         GTTCAGCAAACTC-CTCTCCA-TGGAGCCTGACCGCG-----G-----TCT---------
Methanobacterium_sp._1_452_JQ917190.1/1-452                          GAGCATCCAACTCTCTCTCCA-TCAGGAGTGACGAAG-----G-----TCT---------
Methanobrevibacter_smithii_1_423_GU385700.1/1-423                    GTGCAGCTAACGTATTCTCCA-TCAGAAATT-TGAAG-----A-----T-----------
Methanobrevibacter_oralis_1_414_LK054628.1/1-414                     GTGCTGCTAACGTATTCTCTA-TCAGAAACGATGAAG-----G-----TAT---------
Methanobrevibacter_ruminantium/1-470                                 GTGCAGCTAACGTATTCGCAA-TCAGAAACGACGAAG-----G-----TTT---------
Methanobrevibacter_sp._D5_mcrA_plasmid_365_874_KF214818.1/1-488      GTGCAGCTAACGTATTCTCCA-TCAGAAACGATGAAG-----G-----ATT---------
Methanobrevibacter_gottschalkii_1_471_EU919431.1/1-471               GTGCTGCTAACGTATTCTCCA-TCAGAAATGATGAAG-----G-----TTT---------
Methanobrevibacter_boviskoreani_1_424_KC865050.1/1-424               GTGCTGCTAACGTATTCTCTA-TAAGAAACGATGAAG-----G-----TTT---------
Methanobrevibacter_woesei_1_467_EU919432.1/1-467                     GTGCTGCTAACACATTCTCCT-TCAGAAATGATGAAG-----G-----TTT---------
```

```
Methanobrevibacter_wolinii_1_395_KC865051.1/1-395                   GTGCTGCTAACACATTCTCCT-TCAGAAATGATGAAG-----G-----TTT---------
Methanobrevibacter_arboriphilus_1_414_AF414035.1/1-414              GTGCATCCAACGTATTCTCTA-TAAGAAATGACGAAG-----G-----ATT---------
Methanobacterium_movens_673_1133_HM802934.1/1-445                   GTGCATCTAACGTATTCTCTA-TACGAAACGATGAAG-----G-----ATT---------
Methanobacterium_thermaggregans_1_440_AY289750.1/1-440              GTGCATCCAACGTCTTCTCAA-TAAGGGGCGACGAGG-----G-----ACT---------
Methanothermobacter_wolfeii_259_719_AB300780.1/1-445                GTGCATCCAACGTCTTCTCAA-TAAGGGGCGACGAGG-----G-----ACT---------
Methanothermobacter_tenebrarum_259_723_AB523786.1/1-449             GTGCAGCTAACGTCTTCGCAA-TAAGAGGAGACGAAG-----G-----CCT---------
Methanothermobacter_crinale_18_503_HQ283274.1/1-470                 GTGCAGCCAACGTCTTCGCAA-TAAGAGGAGACGAAG-----G-----ACT---------
Methanobacterium_aggregans_690_1116_KP006500.1/1-411                GTGCATCCAACGTATTCTCCA-TCAGA---------------------------------
Methanobacterium_aarhusense_650_1124_AY386125.1/1-459               GTGCATCCAACGTATTCTCAA-TTAGAGGAGATGAAG-----G-----ACT---------
Methanobacterium_bryantii_259_719_AF313806.1/1-445                  GTGCTGCAAACACATTTGCTA-TCCGTGGAGACGAAG-----G-----ATT---------
Methanococcus_voltae_5430_5930_X07793.1/1-485                       GTGCTTCAAACTCCTTAGCAA-TAAGAAACGATGAAG-----C-----TTC---------
Methanothermococcus_thermolithot_1_414_AF414048.1/1-414             GAGCTTCAAACTCACTTTCAA-TTAGAAACGACGAAG-----C-----TTC---------
Methanococcus_maripaludis_684_1144_AB703637.1/1-445                 GTGCTTCAAACTCACTCGCAA-TCAGAAACGACGAAG-----C-----TGC---------
Methanococcus_vannielii_4514_5014_M16893.1/1-485                    GTGCTTCAAACTCACTCGCAA-TCAGAAACGACGAAT-----C-----TGC---------
Methanococcus_aeolicus_1_464_AY354034.1/1-464                       GAGCTTCAAACTCATTATCTA-TTAGAAATGACGAAT-----C-----CTC---------
Methanosphaera_sp._1_464_FJ982887.1/1-464                           GATCATCCAACTCACTCTCCG-TAGGAAGTGACGAAGGACTTA-----TTC---------
Methanosphaera_stadtmanae_1_414_AF414047.1/1-414                    GATCATCCAACTCACTCTCTG-TAAGAAGTGATGAAGGACTTA-----TTC---------

Methanopyrus_sp._18_510_KC618341.1/1-477                            ----GCCCATGGAGCTCAGGGGACCTAACTACCCCAACTACGCAATGAACGTA-------
Methanomassiliicoccus_luminyensis_1_456_strain_B10_HQ896500.1/1-455 ----GCCCTTCGAGCTCAGGGGCCCCAACTACCC--------------------------
Methanopyrus_kandleri_1_414_AF414042.1/1-414                        ----GCCGCTCGAGCTGCGTGGTCCGAACTAC----------------------------
Methanosarcina_mazei_MH004454.1_RCompl_1_433/1-433                  ----CCCAGACGAACTCCGTGG----------TCCA------------------------
Methanosarcina_horonobensis_256_737_AB288266.1/1-466                ----CCCAGACGAACTCCGTGG----------TCCAA-----------------------
Methanosarcina_subterranea_259_740_AB288268.1/1-466                 ----CCCAGACGAACTCCGTGG----------TCCAA-----------------------
Methanolobus_oregonensis_1_490_U22242.1/1-474                       ----GGCACTTGAACTGCGTGGACCAAACTA-TCCA------------------------
Methanolobus_zinderi_256_745_EU715818.1_RCompl/1-474                ----GGCAGTTGAACTCCGTGGACCAA-CTA-CCCAA-----------------------
Methanolobus_taylorii_1_490_U22243.1/1-474                          ----GGCTCTTGAACTGCGTGGACCAAACTA-TCCA------------------------
Methanolobus_profundi_687_1168_AB703629.1/1-466                     ----TGCTCTTGAACTCCGTGGACCAA---------------------------------
Methanolobus_vulcani_1_490_U22245.1/1-474                           ----TGCTCTTGAACTGCGTGGACCAAACTA-TCCA------------------------
Methanocella_conradii_1_465_JN081865.1/1-450                        ----GCGAGTTGA-----GGGGCCCGAACTA-CCCG------------------------
Methanocella_sp._279_692_KJ441441.1/1-398                           ------------------------------------------------------------
Methanocella_paludicola_240_681_AB300467.1/1-426                    ------------------------------------------------------------
Methanomicrobium_mobile_1_438_AF414044.1/1-438                      ----GATCGGAGAACTTCGCGGACCGAACTA-C---------------------------
Methanoculleus_marisnigri_RCompl_1_436_MH004450.1/1-436             ----AGTCGGAGAACTCCGTGGACCC----------------------------------
Methanoculleus_palmolei_259_743_AB300784.1/1-469                    ----GATCGGCGAGGTCCGCGGCCCGA---------------------------------
Methanoculleus_bourgensis_259_743_AB300787.1/1-469                  GTCTAATGGGCGAGCTGCGTGGTCCGA---------------------------------
Methanoculleus_chikugoensis_259_743_AB300779.1/1-469                GTCTGATGGGAGAACTGCGTGGTCCGA---------------------------------
Methanogenium_organophilum_1_501_DQ229160.1/1-487                   ----CATGGGCGAACTGCGTGGACCTAACTA-TCCGACTACGCCATGA------------
Methanogenium_boonei_15_520_DQ229161.1/1-490                        ----GATGGGCGAACTCCGTGGACCAAACTA-TCCGACTACGCCATGAA---A-------
Methanogenium_cariaci_1_433_DQ229157.1/1-420                        ----GATGGGCGAACTCT-TGGACCAA-CTA-TCCAACTACGCCATGA------------
Methanobacterium_sp._1_452_JQ917190.1/1-452                         ----GATCCACGAACTACGTGGTCCTAACTATCCCAACTACGCCATGAA---C-------
Methanobrevibacter_smithii_1_423_GU385700.1/1-423                   ------------------------------------------------------------
Methanobrevibacter_oralis_1_414_LK054628.1/1-414                    ----ACCACTTGAAATGAGAGGCCCT----------------------------------
Methanobrevibacter_ruminantium/1-470                                ----ACCACTTGAATTAAGAGGACCTAACTATCCTAACTATGCAATGAA-----------
Methanobrevibacter_sp._D5_mcrA_plasmid_365_874_KF214818.1/1-488     ----ACCACTCGAAATGAGAGGACCAAACTATCCTAACTACGCAATGAA---CGTGGTAC
Methanobrevibacter_gottschalkii_1_471_EU919431.1/1-471              ----ACCACTCGAAATGAGAGGACCAAACTACCCAAACTACGCAATGAA-----------
Methanobrevibacter_boviskoreani_1_424_KC865050.1/1-424              ----ACCACTTGAAATGAGAGGTCCTAACTACCCTAACTACGCAATGAA-----------
Methanobrevibacter_woesei_1_467_EU919432.1/1-467                    ----ACCTCTTGAAATGAGAGGACCTAACTACCCTAACTACGCAATGAA-----------
Methanobrevibacter_wolinii_1_395_KC865051.1/1-395                   ----ACCTCTTGAAATGAGAGGACCTAACTATCCTAACTACGCAATGAA-----------
Methanobrevibacter_arboriphilus_1_414_AF414035.1/1-414              ----ACCAGTTGAAATGAGAGGGCCAAACTAC----------------------------
Methanobacterium_movens_673_1133_HM802934.1/1-445                   ----ACCACTCGAAATGAGAGGACCAA---------------------------------
Methanobacterium_thermaggregans_1_440_AY289750.1/1-440              ----GCCACTGGAGCTTCGTGG--------------------------------------
Methanothermobacter_wolfeii_259_719_AB300780.1/1-445                ----GCCACTGGAGCTTCGTGGA-CCTA--------------------------------
Methanothermobacter_tenebrarum_259_723_AB523786.1/1-449             ----ACCATTAGAGGCAAGGGGAGCCAA--------------------------------
Methanothermobacter_crinale_18_503_HQ283274.1/1-470                 ----ACCATTAGAGGCAAGAGGAGCCAATTATCCAAACTACGCAATGAA---CGT-----
Methanobacterium_aggregans_690_1116_KP006500.1/1-411                ------------------------------------------------------------
Methanobacterium_aarhusense_650_1124_AY386125.1/1-459               ----ACCACTAGAACTTAGAGGAGCAAACTACCCCAACTA--------------T-----
Methanobacterium_bryantii_259_719_AF313806.1/1-445                  ----ACCTCTTGAAGCAAGAGGAGCAA---------------------------------
Methanococcus_voltae_5430_5930_X07793.1/1-485                       ----ACCATTAGAATTAAGAGGTCCTAACTACCCTAACTATGCAATGAA---CGT-----
Methanothermococcus_thermolithot_1_414_AF414048.1/1-414             ----ACCATTGGAATTAAGAGGTCCAAACTAC----------------------------
Methanococcus_maripaludis_684_1144_AB703637.1/1-445                 ----ACCTCTTGAATTAAGAGGACCTA---------------------------------
Methanococcus_vannielii_4514_5014_M16893.1/1-485                    ----ACCTCTTGAATTAAGGGGGCCTAACTACCCTAACTACGCAATGAA---CGT-----
Methanococcus_aeolicus_1_464_AY354034.1/1-464                       ----ACCATTGGAATTAAGAGGTCCAAACTACCCTAACTACGCAATG-------------
Methanosphaera_sp._1_464_FJ982887.1/1-464                           ----AC-----GAATTAAGAGGTCCTAACTATCCTAACTATGCAATG-------------
Methanosphaera_stadtmanae_1_414_AF414047.1/1-414                    ----AC-----GAATTAAGAGGTCCTAACTAC----------------------------

Methanopyrus_sp._18_510_KC618341.1/1-477                            ------------------------------
Methanomassiliicoccus_luminyensis_1_456_strain_B10_HQ896500.1/1-455 ------------------------------
Methanopyrus_kandleri_1_414_AF414042.1/1-414                        ------------------------------
Methanosarcina_mazei_MH004454.1_RCompl_1_433/1-433                  ------------------------------
Methanosarcina_horonobensis_256_737_AB288266.1/1-466                ------------------------------
Methanosarcina_subterranea_259_740_AB288268.1/1-466                 ------------------------------
Methanolobus_oregonensis_1_490_U22242.1/1-474                       ------------------------------
Methanolobus_zinderi_256_745_EU715818.1_RCompl/1-474                ------------------------------
Methanolobus_taylorii_1_490_U22243.1/1-474                          ------------------------------
Methanolobus_profundi_687_1168_AB703629.1/1-466                     ------------------------------
Methanolobus_vulcani_1_490_U22245.1/1-474                           ------------------------------
Methanocella_conradii_1_465_JN081865.1/1-450                        ------------------------------
Methanocella_sp._279_692_KJ441441.1/1-398                           ------------------------------
Methanocella_paludicola_240_681_AB300467.1/1-426                    ------------------------------
Methanomicrobium_mobile_1_438_AF414044.1/1-438                      ------------------------------
Methanoculleus_marisnigri_RCompl_1_436_MH004450.1/1-436             ------------------------------
Methanoculleus_palmolei_259_743_AB300784.1/1-469                    ------------------------------
Methanoculleus_bourgensis_259_743_AB300787.1/1-469                  ------------------------------
Methanoculleus_chikugoensis_259_743_AB300779.1/1-469                ------------------------------
Methanogenium_organophilum_1_501_DQ229160.1/1-487                   ------------------------------
Methanogenium_boonei_15_520_DQ229161.1/1-490                        ------------------------------
Methanogenium_cariaci_1_433_DQ229157.1/1-420                        ------------------------------
Methanobacterium_sp._1_452_JQ917190.1/1-452                         ------------------------------
Methanobrevibacter_smithii_1_423_GU385700.1/1-423                   ------------------------------
Methanobrevibacter_oralis_1_414_LK054628.1/1-414                    ------------------------------
Methanobrevibacter_ruminantium/1-470                                ------------------------------
Methanobrevibacter_sp._D5_mcrA_plasmid_365_874_KF214818.1/1-488     CTGGAGCACA--------------------
Methanobrevibacter_gottschalkii_1_471_EU919431.1/1-471              ----------A-------------------
Methanobrevibacter_boviskoreani_1_424_KC865050.1/1-424              -----------AA-----------------
Methanobrevibacter_woesei_1_467_EU919432.1/1-467                    ------------------------------
Methanobrevibacter_wolinii_1_395_KC865051.1/1-395                   -----------A------------------
Methanobrevibacter_arboriphilus_1_414_AF414035.1/1-414              ------------------------------
Methanobacterium_movens_673_1133_HM802934.1/1-445                   ------------------------------
Methanobacterium_thermaggregans_1_440_AY289750.1/1-440              ------------------------------
Methanothermobacter_wolfeii_259_719_AB300780.1/1-445                ------------------------------
Methanothermobacter_tenebrarum_259_723_AB523786.1/1-449             ------------------------------
Methanothermobacter_crinale_18_503_HQ283274.1/1-470                 ------------------------------
Methanobacterium_aggregans_690_1116_KP006500.1/1-411                ------------------------------
```

```
Methanobacterium_aarhusense_650_1124_AY386125.1/1-459              ------------------------------
Methanobacterium_bryantii_259_719_AF313806.1/1-445                 ------------------------------
Methanococcus_voltae_5430_5930_X07793.1/1-485                      -------------AGGTCACCAAGGTGA--
Methanothermococcus_thermolithot_1_414_AF414048.1/1-414            ------------------------------
Methanococcus_maripaludis_684_1144_AB703637.1/1-445                ------------------------------
Methanococcus_vannielii_4514_5014_M16893.1/1-485                   -------------AGGTCACCAGGGAGA--
Methanococcus_aeolicus_1_464_AY354034.1/1-464                      ------------------------------
Methanosphaera_sp._1_464_FJ982887.1/1-464                          ----------------------------AA
Methanosphaera_stadtmanae_1_414_AF414047.1/1-414                   ------------------------------
```
