# Supplementary material for: Improved Quantitative Real-Time PCR Protocol for Detection and Quantification of Methanogenic Archaea in Stool Samples
Source: Microorganisms. 2023 Mar 5;11(3):660. doi: 10.3390/microorganisms11030660 (PMC10051802; doi:10.3390/microorganisms11030660)
Supplement: Supplementary file 1 [file microorganisms-11-00660-s001.zip › Table S2.pdf]

CLUSTAL

```
gi309253887/1-472 GGTGGCGTCGGATTCACAATGTATGCAACACCAGCATACACCAATGATATCGTCGATGAC
gi930068978/1-469 GGTGGTGTCGGGTTCACACAGTATGCAACCGCAGCATACACCGACAACATCCTCGATGAC
gi930068946/1-466 GGTGGTGTCGGGTTCACACAGTATGCAACCGCAGCATACACCGACAACATCCTCGACGAC
gi954036668/1-468 GGTGGTGTCGGATTCACCCAGTATGCAACCGCTGCATACACCGACAACATCCTTGACGAC
gi930068996/1-469 GGTGGTGTCGGATTCACCCAATATGCAACCGCTGCATACACCGACAACATCCTCGATGAC
gi428274410/1-489 GGTGGTGTCGGATTCACCCAGTATGCAACAGCCGCATACACCGACAATATCCTCGATGAG
gi954036662/1-94  GGTGGTGTCGGCTTTACCCAGTATGCAACAGCTGCATACACCGACGATATCCTCGACAAC
gi954036666/1-486 GGTGGTGTCGGCTTTACCCAGTATGCAACAGCTGCATACACCGACGATATCCTCGACAAC
gi930068992/1-487 GGTGGTGTCGGCTTTACCCAGTATGCAACAGCTGCATACACTGACGATATCCTCGACAAC
gi930068990/1-487 GGTGGTGTCGGGTTCACTCAGTATGCAACAGCTGCATACACTGATGATATCCTCGACAAC
gi950655841/1-488 GGTGGCGTCGGGTTCACCCAGTATGCAACAGCTGCATACACCGATGACATCCTCGACAAC
gi930068952/1-487 GGTGGTGTCGGGTTCACTCAGTATGCAACAGCTGCATACACTGATGACATCCTTGACAAC
gi58615486/1-470  GGTGGTGTCGGATTCACGCAGTACGCAACAGCAGCATACACCAACGATGTGCTGGATGAC
gi5616127/1-466   GGTGGTGTCGGATTCACGCAGTACGCAACAGCGGCATACACGAACGATGTCCTGGATGAC
gi82492006/1-464  GGTGGTGTAGGATTCACACAATATGCTACAGCAGCATATACTGATAATGTATTAGATGAC
gi262358095/1-470 GGTGGTGTAGGATTCACACAGTATGCAACAGCAGCATATACTGATAATGTATTAGATGAC
gi629633272/1-472 GGTGGTGTAGGATTCACACAATATGCTACTGCAGCATACACTGATAATGTATTAGACGAC
gi629633282/1-472 GGTGGTGTAGGATTCACTCAATATGCTACTGCAGCATATACTGATAATGTATTAGACGAC
gi334085683/1-468 GGTGGTGTAGGATTCACCCAATACGCAACCGCAGCATACACTGACGACGTATTAGATGAC
gi217792440/1-467 GGTGGTGTAGGATTCACACAGTATGCTACAGCAGCATATACTGATAATGTTCTTGATGAC
gi498543114/1-395 ------------------------------------------------------------
gi629633278/1-472 GGTGGTGTAGGATTTACTCAATATGCTACCGCAGCATATACTGATAATGTATTAGATGAC
gi145370874/1-445 GGTGGTGTTGGATTTACTCAATACGCATCTGCAGCTTATACTGACAATATTCTTGACGAT
gi311141403/1-440 GGTGGTGTTGGATTTACCCAATACGCATCTGCAGCTTATACTGACAATATTCTTGACGAT
gi629633284/1-466 GGTGGTGTTGGTTTTACACAATATGCTAGTGCAGCTTATACTGATGATATCCTTGACGAT
gi629633280/1-466 GGTGGTGTAGGATTTACTCAATATGCAAGTGCAACATATACTGATGATATATTAGATGAT

gi309253887/1-472 TTCCTGTACTGGGGCAACGACTATGCAGCAAAGAA--ATA---CGGC-----GG------
gi930068978/1-469 TACTGCTACTACGGTATGGACTATATCAAGTCCAA--GTT---CGGT-----GG------
gi930068946/1-466 TACACCTACTACGGTATGGACTATGTCAAGTCCAA--GTA---CGGT-----GG------
gi954036668/1-468 TACTGCTACTATGGTCTTGACTATGTCAAGAAGAA--CCA---CGGT-----GG------
gi930068996/1-469 TACTGTTATTACGGTCTTGACTATGTCAAGAAGAA--ACA---CGGT-----GG------
gi428274410/1-489 TTCTGCTACTATGGTATGGACTACATCAAAGACAAGTACA---AGGTCGACTGG------
gi954036662/1-94  AGCGTGTACTACAACATCGACTACATCAACGACA--------------------------
gi954036666/1-486 AACGTGTACTACAACATCGACTACATCAACGACAA--GTA---CAAA-----GGTGCTGC
gi930068992/1-487 AACGTGTACTACAATGTCGACTACATCAACGACAA--GTA---CAAA-----GGTGCTGC
gi930068990/1-487 AACGTGTACTACAACGTCGACTACATCAACGACAA--GTA---CAAG-----GGTGCTGC
gi950655841/1-488 AACACCTACTACAACGTTGACTACATCAACGACAA--GTA---CAAC-----GGTGCTGC
gi930068952/1-487 AACGTGTACTATGATGTAGACTACATCAACCAGAA--GTA---CAAC-----AATGCTGC
gi58615486/1-470  TTCACGTACTACGGATACGACTACGCACTGAACAA--GTA---CGGA-----GA------
gi5616127/1-466   TTCACGTACTACGGATACGACTACGCACTGAAGAA--GTA---CGGA-----GA------
gi82492006/1-464  TTCACTTACTTCGGTAAAGATTATGTTGAAGATAA--ATACGG---T-----GG------
gi262358095/1-470 TTTACTTATTATGGTAAAGCTTATGTCGAAGCTAA--ATATGG---A-----GA------
gi629633272/1-472 TTCACCTACTACGGTAAAGACTACGTCGAAGACAA--ATACGG---A-----GA------
gi629633282/1-472 TTCACCTACTATGGTAAAGACTACGTCGAAGACAA--ATACGG---C-----GA------
gi334085683/1-468 TTCACCTACTACGGTAAAGACTACGTAGAAGACAA--ATACGG---T-----GG------
gi217792440/1-467 TTTGCTTACTATGGTAAAGATTATGTAGAAGATAA--ATATGG---T-------------
gi498543114/1-395 -------------GTAAAGATTATGTAGAAGATAA--ATATGG---T-------------
gi629633278/1-472 TTCTCTTACTTCGGTAAAGATTATGTAGAAGACAA--ATACGG---T-----GG------
gi145370874/1-445 TTCTTATACTATGGTAAAGAATATGTCGAAGACAA--ATT------T-----GG------
gi311141403/1-440 TTCTTATACTATGGTAAAGAATATGTCGAAGACAA--ATT------T-----GG------
gi629633284/1-466 TTCATTTATTATGGAATGGATTATGTCGAAAAGAA--TTA------T-----GG------
gi629633280/1-466 TTTATTTACTATGGAAAAGATTATGTAGAGAAAAA--TTA------T-----GG------

gi309253887/1-472 ---AA--------------ATGG---------AAAGGCA------AAAGCCAC-------
gi930068978/1-469 ---TC--------------TCGG---------CAAAGTTCCATGCAAG------------
gi930068946/1-466 ---AG--------------CCGG---------CAAGGTACCATGCAAG------------
gi954036668/1-468 ---CA--------------TCGG---------CAAGGCA------AAGCACAC-------
gi930068996/1-469 ---CA--------------TCGG---------AAAGGCA------AAGTCAAC-------
gi428274410/1-489 ---CAGAACCCGAATCCAAACGA---------CAAGGTC------AAGCCAAC-------
gi954036662/1-94  ------------------------------------------------------------
gi954036666/1-486 AAATG--------------TCGGCAAGGACAACAAGGTA------AAGGCAAC-------
gi930068992/1-487 AAATA--------------TAGGCAAGGACAATAAGGTA------AAGGCAAC-------
gi930068990/1-487 AAACA--------------TCGGCACGGACAACAAGGTA------AAAGCAAC-------
gi950655841/1-488 AAACG--------------TCGGTAAGGACAACAAGGTA------AAGGCAAC-------
gi930068952/1-487 AACAG--------------TCGGCAAGGACAACAAGGTA------AAGGCAAC-------
```

gi58615486/1-470 ---AG--------------ACGG---------------------------AACCGCACCA
gi5616127/1-466   ---AG--------------ACGG---------------------------AACAGCACCA
gi82492006/1-464 ---AT--------------TAAC---------TCAAGCA------CCAAATAA-------
gi262358095/1-470 ---GT--------------TATG---------TGCAGCA------CCTAACAA-------
gi629633272/1-472 ---AT--------------TATG---------CGCAGCA------CCTAACAA-------
gi629633282/1-472 ---CT--------------TATG---------TTCCGCA------CCTAACAA-------
gi334085683/1-468 ---AT--------------TAAC---------CGAAGCA------CCTAACAA-------
gi217792440/1-467 ----T--------------TAAC---------CAAAGCA------CCTAACAA-------
gi498543114/1-395 ----T--------------TAAC---------CAAAGCA------CCTAACAA-------
gi629633278/1-472 ---AT--------------TATG---------TGAAGCT------CCAAACAA-------
gi145370874/1-445 ---AA--------------TATG---------TGAAGCT------CCAAACAA-------
gi311141403/1-440 ---AA--------------TATG---------TGAAGCT------CCAAACAA-------
gi629633284/1-466 ---GA--------------TGTG---------TAATGCT------GAATTAGA-------
gi629633280/1-466 ---AA--------------TGTG---------TCAGGCA------GAAGCTAA-------

gi309253887/1-472 -----CATTGACACTGTAAAGGATATCGCTACAGAAACAACTCTGTACGGTCTTGAAGCA
gi930068978/1-469 ------CAGGACGCAGTCAACGATGTTGCAACTGAAGTCACCCTCTACGGTATGGAACAG
gi930068946/1-466 ------CAGGACGCAGTCAACGATGTTGCAACTGAAGTCACGCTCTATGGTATGGAACAG
gi954036668/1-468 -----CCAGGAAGTTATCAACGACATTGCAACCGAAACCACGCTCTACGGTATGGAACAG
gi930068996/1-469 -----CCAGGAAGCAGTTACTGACATTGCAACCGAGGCTAACCTCTACGGTATGGAACAG
gi428274410/1-489 -----CCAGGAAGTCGTCAACGACATTGCAAGCGAGGTCAACCTCAACGGTATGGAACAG
gi954036662/1-94 --
gi954036666/1-486 -----CCTCGAAGTCGTAAAGGACATTGCAACCGAGTCCACACTCTACGGTATCGAGACC
gi930068992/1-487 -----CCTCGACGTCGTAAAGGACATTGCAACCGAGTCCACACTCTACGGTATCGAGACC
gi930068990/1-487 -----TCTCGAAGTCGTAAAGGACATTGCAACTGAGTCCACAATCTACGGTATCGAGACC
gi950655841/1-488 -----CCTCGACGTCGTAAAGGACATCGCAACCGAATCCACAATCTACGGTATCGAGACC
gi930068952/1-487 -----CCTCGAAGTCGTAAAGGACATCGCAACCGAGTCCACAATCTACGGTATCGAGACA
gi58615486/1-470 AACGACCTTGCAACCGCAACCGACCTTGCAACCGAGGTTACCTTAAACGCTATGGAGTGC
gi5616127/1-466   AACGACCTTGCAACCGCAACCGACCTTGCAACCGAGGTTACCTTGAACGGTATGGAGTGC
gi82492006/1-464 -----TATGGATACTGTTTTAGATGTTGGTACTGAAGTTGCATTCTATGCATTAGAACAA
gi262358095/1-470 -----CATGGATACTGTACTTGATGTAGGTACTGAAGTAGCATTCTACGCATTAGAACAA
gi629633272/1-472 -----CATGGACACCGTTCTTGATGTAGGTACTGAAGTAGCATTCTACGCATTAGAACAA
gi629633282/1-472 -----CATGGACACTGTTTTAGATGTTGGTACTGAAGTAGCATTCTACGCATTAGACCAA
gi334085683/1-468 -----CATGGACACTGTTCTTGATGTAGGTACTGAAGTTACTTTCTACTCCTTAGAACAA
gi217792440/1-467 -----CATGGACACTGTTCTTGATGTTGGTTCTGAAGTTACCTTCTACGGATTAGAACAA
gi498543114/1-395 -----CATGGACACTGTTCTTGATGTTGGTTCTGAAGTTACCTTCTACGGATTAGAACAA
gi629633278/1-472 -----CATGGACACTGTTCTTGATGTAGGTTCTGAAGTAGCATTCTACTCCTTAGAACAA
gi145370874/1-445 -----CATGGACACTGTTCTTGATGTAGGTTCTGAAGTAACTTTCTATGGATTAGAACAA
gi311141403/1-440 -----CATGGACACTGTTCTTGATGTAGGTTCTGAAGTAACTTTCTATGGATTAGAACAA
gi629633284/1-466 -----TATGGATACTGTACGTGATATATCTACTGAAGTAACCCTTTATGGTTTAGAACAG
gi629633280/1-466 -----CTTTGATAATGTAAAGGATATTGCTACTGAAGTTACAATTTATGGTTTAGAACAG

gi309253887/1-472 TACGAGAAATACCCAACCACA**CTGGAAGACCATTTCGGTGGTTC**ACAGAGAGCAACAGTC
gi930068978/1-469 TACGAGCAGTACCCGACTGCA**CTCGAGAGCCACTTCGGTGGGTC**ACAGCGTGCATCCGTC
gi930068946/1-466 TACGAGCAGTACCCGACTGCA**CTCGAGAGCCACTTCGGTGGGTC**ACAGCGTGCATCCGTA
gi954036668/1-468 TACGAACAGTACCCGACCACC**CTCGAGAGCCACTTCGGCGGTTC**CCAGCGTGCATCCGTC
gi930068996/1-469 TACGAACAGTACCCGACCGCA**CTCGAGAGCCACTTCGGCGGTTC**CCAGCGTGCCTCCGTC
gi428274410/1-489 TACGAACAGTACCCGACCATG**CTTGAAGACCACTTCGGTGGCTC**ACAGCGTGCAAGTGTC
gi954036662/1-94 --
gi954036666/1-486 TACGAAAAGTTCCCAACTGCC**CTTGAAGACCACTTCGGTGGGTC**CCAGAGAGCAACCGTG
gi930068992/1-487 TACGAAAAGTTCCCAACTGCC**CTTGAAGACCACTTCGGTGGATC**CCAGAGAGCAACCGTG
gi930068990/1-487 TACAAGAAGTTCCCGACTGCC**CTTGAAGACCACTTCGGTGGATC**CCAGAGAGCAACCGTG
gi950655841/1-488 TACGAGAAGTTCCCGACTGCC**CTTGAAGACCACTTCGGAGGTTC**CCAGAGAGCAACCGTG
gi930068952/1-487 TACGAGAAGTTCCCAACAGCC**CTTGAAGACCACTTCGGTGGTTC**CCAGAGAGCAACCGTG
gi58615486/1-470 TACGAGGACTACCCAACGCTT**CTTGAGGACCACTTCGGTGGATC**GCAGCGTGCAGGTGTC
gi5616127/1-466   TACGAGGACTACCCAACGCTT**CTTGAGGACCACTTCGGTGGATC**GCAGAGAGCGGGAATC
gi82492006/1-464 TACGAAGAATACCCAGCATTA**CTCGAAACTCACTTCGGTGGATC**TCAAAGATCATCTGTT
gi262358095/1-470 TATGAAGAATATCCGGCATTA**CTCGAAACTCACTTTGGCGGATC**TCAAAGATCTTCAGTT
gi629633272/1-472 TACGAAGAATACCCAGCTTTA**CTTGAAACTCACTTCGGTGGATC**TCAAAGAGCTTCCGTT
gi629633282/1-472 TACGAAGAATACCCAGCTTTA**CTTGAAACCCACTTCGGTGGATC**TCAAAGAGCTTCCGTT
gi334085683/1-468 TACGAAGAATACCCAGCTTTA**CTCGAAACTCACTTCGGTGGATC**TCAAAGAGCTTCCGTT
gi217792440/1-467 TATGAAAAATTCCCTGCATTA**CTTGAAACTCAATTCGGTGGATC**CCAAAGGGCTGCTGTT
gi498543114/1-395 TATGAAAAATTCCCTGCATTA**CTTGAAACTCAATTCGGTGGATC**CCAAAGGGCTGCTGTT
gi629633278/1-472 TACGAAGAATACCCAGCTTTA**CTTGAAACCCACTTCGGTGGTTC**TCAAAGAGCTGCTGTT
gi145370874/1-445 TACGAAGAATATCCTGCATTA**CTTGAAACCCAATTCGGTGGTTC**TCAAAGAGCATCTGTT
gi311141403/1-440 TACGAAGAATATCCTGCATTA**CTTGAAACCCAATTCGGTGGTTC**TCAAAGAGCATCTGTT
gi629633284/1-466 TATGAG--ATACCTA-CTCTT**TTAGAAACTCACTTTGGTGGTTC**TCAAAGAGCATCAGTT
gi629633280/1-466 TACGAA--ATCCCAA-CATTA**CTTGAAGACCATTTCGGTGGCTC**TCAAAGAGCATCGGTT

```
gi309253887/1-472 ATATCAATTGCAGCAGGCG---GCGCAACAGCTCT------G------------GCAACA
gi930068978/1-469 CTTGCC---GCAG------------CAGCAGGTCT------GACCACCTCACTCGGTACC
gi930068946/1-466 CTTGCA---GCAG------------CATCAGGTCT------GTCCTGTTCACTCGGTACC
gi954036668/1-468 CTTGCA---GCAG------------CATCAGGTAT------CTCCTGTTCACTCGCAACC
gi930068996/1-469 CTTGCA---GCAG------------CATCAGGTAT------CTCCTGTTCACTGGCAACT
gi428274410/1-489 CTCGCA---GCAG------------CATCCGGTAT------CTCCACCTCAATTGCAACC
gi954036662/1-94  ------------------------------------------------------------
gi954036666/1-486 CTTGCA---GCAG------------CAGCCGGTGTCTGTACTGCA------ATCGCAACC
gi930068992/1-487 CTCGCA---GCTG------------CAGCCGGTGT------TGCATGTTCCCTTGCAACC
gi930068990/1-487 CTCGCA---GCCG------------CAGCCGGTGT------CGCATGTTCCCTCGCAACC
gi950655841/1-488 CTCGCA---GCCG------------CAGCCGGTGT------CGCAACTGCCCTCGCAACC
gi930068952/1-487 CTCGCA---GCCG------------CAGCCGGTGT------TGCATGCGCCCTCGGAACT
gi58615486/1-470  ATGGCA---GCAGCAAGCGCATGCACAACCGGTAT------T------------GCTACC
gi5616127/1-466   CTTGCA---GCAGCAAGCGCATGCACAACCGGTAT------T------------GCTACC
gi82492006/1-464  ATTTCA---GCAGCAGCTGGTGCTTCAACTGCATT------T------------GCTACT
gi262358095/1-470 ATTTCA---GCAGCAGCTGGCTGTTCCACTGCATT------C------------GCTACT
gi629633272/1-472 GTTTCC---GCAGCTGCAGGTTGTTCCACTGCATT------C------------GCTACT
gi629633282/1-472 ATTTCC---GCAGCTGCTGGTTGTTCCACTGCATT------C------------GCTACT
gi334085683/1-468 GTTTCC---GCAGCTGCTGGTTGTTCCACTGCATT------C------------GCTACT
gi217792440/1-467 GTTGCA---GCTGCTGCAGGTATTTCAACTGCATT------T------------GCTACT
gi498543114/1-395 GTTGCA---GCTGCTGCAGGTATTTCAACTGCATT------T------------GCTACT
gi629633278/1-472 ATTTCC---GCAGCTTCCGGTATTTCCACTGCATT------T------------GCAACA
gi145370874/1-445 GTTTCA---GCCGCTGCTGGTTGTGCAACAGCTTT------C------------GCTACT
gi311141403/1-440 GTTTCA---GCCGCTGCTGGTTGTGCAACAGCTTT------C------------GCTACT
gi629633284/1-466 ATTTCC---GCCGCTGCAGGTTGTTCAACTGCTTT------T------------GCAACA
gi629633280/1-466 ATTTCC---GCAGCTGCAGGATGTTCTGTAGCATT------T------------GCAACA

gi309253887/1-472 GGCCACAGCCAGGCCGGTCTCTCTGCATGGTACCTGTCCATGTATCCCCACAAAGAGGCA
gi930068978/1-469 GGAAACTCCAATGCCGGTCTGAATGGCTGGTACCTGTCCATGCTCCTGCACAAGGAAGGC
gi930068946/1-466 TGCAACTCGAATGCAGGTCTGCACGGATGGTACATGTCGATGCTCCTGCACAAGGAAGGA
gi954036668/1-468 GGAAACTCGAACGCTGGCCTGAACGGCTGGTACATGTCCATGCTTGCCCACAAGGAAGGC
gi930068996/1-469 GCAAACTCAAATGCAGGCCTGAACGGCTGGTACTTGTCGATGCTTATGCACAAGGAAGGC
gi428274410/1-489 GGCAACTCAAATGCCGGTCTGAACGGCTGGTACCTCTCGATGCTCATGCACAAAGAGGGA
gi954036662/1-94  ------------------------------------------------------------
gi954036666/1-486 GCAAACGCAAATGCCGGTCTCTCCGGCTGGTACCTCTCCATGTACCTGCACAAGGAAGCA
gi930068992/1-487 GCAAACGCAAATGCAGGTCTCTCCGGCTGGTACCTCTCCATGTACCTGCACAAGGAAGCA
gi930068990/1-487 GCAAACGCAAATGCTGGTCTCTCTGGCTGGTACCTCTCCATGTACCTGCACAAGGAAGCA
gi950655841/1-488 TCAAACGCCAATGCCGGACTCTCAGGCTGGTACCTCTCCATGTACCTGCACAAGGAAGCA
gi930068952/1-487 GCAAACGCCAACGCCGGTCTCTCAGGCTGGTACCTTTCCATGTACGTCCACAAGGAAGCA
gi58615486/1-470  GGTAACGCACAGGTCGCGCTCTCCGGATGGTACATGTCGATGTACCTGCACAAGGAAGGC
gi5616127/1-466   GGAAACGCACAGGTCGCACTGTCTGCATGGTGCATGTCGATGTACCTGCACAAGGAAGGC
gi82492006/1-464  GGTAATGCTCAAACTGGTTTAAGTGCATGGTACTTATCTATGTATTTACACAAAGAACAA
gi262358095/1-470 GGTAATGCTCAAACTGGTTTGAGTGCATGGTACTTGTCCATGTACTTGCACAAAGAACAA
gi629633272/1-472 GGTAATGCACAAACTGGTTTAAGCGCATGGTACTTAGCTATGTACTTACACAAAGAACAA
gi629633282/1-472 GGTAATGCACAAACTGGTTTAAGTGCATGGTACTTAGCTATGTACTTACACAAAGAACAA
gi334085683/1-468 GGTTATGCACAAACTGGTTTAAGCGCATGGTACTTAGCTATGTACTTACACAAAGAACAA
gi217792440/1-467 GGAAATGCTCAAACTGGTTTAAGTGCATGGTACTTATCTCAATACTTACACAAAGAACAA
gi498543114/1-395 GGAAATGCTCAAACTGGTTTAAGTGCATGGTACTTATCTCAATACTTACACAAAGAACAA
gi629633278/1-472 GGTAATGCACAAACTGGTTTATCTGCATGGTACTTAGCACAATACTTACACAAAGAACAA
gi145370874/1-445 GGAAACTCCCAAACTGGTTTAAGCGCATGGTATTTATCTATGTACTTACACAAAGAACAA
gi311141403/1-440 GGAAACTCCCAAACTGGTTTAAGCGCATGGTATTTATCTATGTACTTACACAAAGAACAA
gi629633284/1-466 GGAAATTCCAATGCTGGTGTAAATGGATGGTATTTAAGTATGATTTTACATAAAGAAGCT
gi629633280/1-466 GGAAACTCTAATGCAGGAGTAAATGGTTGGTATCTCAGTATGATTGAACATAAAGAAGCT

gi309253887/1-472 CACGGCAGACTCGGATTCTACGGGTATGACCTGCAGGATCAGTGCGGTGCAACGAACGTG
gi930068978/1-469 TGGTCACGTCTCGGCTTCTTCGGCTACRACCTGCAGGACCAGTGTGGTTCCGCAAACTCG
gi930068946/1-466 TGGCCACGTCTCGGCTTCTTCGGCTACAACCTGCAGGACCAGTGCGGTTCCGCAAACTCG
gi954036668/1-468 TGGTCACGTCTCGGCTTCTTCGGTTACGACCTGCAGGACCAGTGCGGTTCAGCAAACTCA
gi930068996/1-469 TGGTCACGTCTCGGCTTCTTCGGCTACGACCTGCAGGACCAGTGCGGTTCCGCAAACACC
gi428274410/1-489 TGGTCACGTCTCGGATTCTTCGGCTACGACCTGCAGGACCAGTGTGGTTCAGCAAACACC
gi954036662/1-94  ------------------------------------------------------------
gi954036666/1-486 TGGGGCCGTCTCGGCTTCTTCGGATACGACCTGCAGGATCAGTGCGGTGCCACAAATGTT
gi930068992/1-487 TGGGGCCGTCTCGGCTTCTTCGGATACGACCTTCAGGATCAGTGCGGTGCCACAAATGTT
gi930068990/1-487 TGGGGCCGTCTCGGCTTCTTCGGATACGACCTGCAGGACCAGTGCGGTGCCACAAATGTT
gi950655841/1-488 TGGGGCAGACTTGGATTCTTCGGATTCGACCTGCAGGACCAGTGCGGTGCTACAAACGTT
gi930068952/1-487 TGGGGCCGTCTCGGCTTCTTCGGTTTCGACCTGCAGGACCAGTGTGGTGCCACAAACGTT
gi58615486/1-470  TGGGGCAGACTCGGATTCTTCGGGTACGACCTGCAAGACCAGTGCGGTGCAACCAACGTC
```

```
gi5616127/1-466   TGGGGACGACTCGGATTCTTCGGATACGACTTGCAGGATCAGTGCGGTGCAACCAACGTC
gi82492006/1-464  CATTCCAGATTAGGTTTCTATGGTTTCGATTTACAAGATCAATGTGGTGCTGCTAACGTA
gi262358095/1-470 CATTCCAGATTAGGATTCTATGGTTTCGATTTACAAGATCAATGTGGTGCAGCTAACGTA
gi629633272/1-472 CACTCCAGATTAGGATTCTACGGTTTCGATTTACAAGACCAATGTGGTGCAGCTAACGTA
gi629633282/1-472 CACTCCAGATTAGGATTCTACGGATACGATTTACAAGATCAATGTGGTGCTGCTAACGTA
gi334085683/1-468 CACTCCAGATTAGGATTCTACGGTTTCGATTTACAAGATCAATGTGGTGCAGCTAACACT
gi217792440/1-467 CATTCCAGATTAGGTTTCTATGGTTACGATTTACAAGATCAATGTGGTGCTGCTAACACA
gi498543114/1-395 CATTCCAGATTAGGTTTCTATGGTTACGATTTACAAGATCAATGTGGTGCTGCTAACACA
gi629633278/1-472 CATTCCAGATTAGGATTCTATGGTTACGACTTACAAGACCAATGTGGTGCAGCTAACGTA
gi145370874/1-445 CACTCTAGATTAGGATTCTATGGTTACGATTTACAAGATCAATGTGGTGCATCCAACGTA
gi311141403/1-440 CACTCTAGATTAGGATTCTATGGTTACGATTTACAAGATCAATGTGGTGCATCCAACGTA
gi629633284/1-466 CATTCCAGACTTGGATTTTATGGATATGATTTACAAGACCAAGCAGGTGCTTCTAATTCA
gi629633280/1-466 CACAGCAGATTAGGATTCTATGGTTACGATTTACAAGACCAATGTGGTGCAGCTAATTCA

gi309253887/1-472 TTCTCAATTGCATCA----GATGAAG-----GC--------TGTGTAGGCGAATGCGGAG
gi930068978/1-469 ATGGC----CATCAAAGGCGACGAGG-----GT--------TGTATCGGAGAACTCCGTG
gi930068946/1-466 ATGGC----CATCAAAGGCGACGAGG-----GT--------TGTATCGGAGAACTCCGTG
gi954036668/1-468 ATGTC----CGTCCGGCCCGACGAGG-----GC--------TGTATCGGCGAGCTCCGTG
gi930068996/1-469 GAGTC----CTACAGGGCAGACGAAG-----GC--------TGTGTCGGAGAGCTCCGTG
gi428274410/1-489 CTCTC----TGTCCGGCCTGACGAAG-----GC--------TGTATCGGCGAGTTCCGTG
gi954036662/1-94  ------------------------------------------------------------
gi954036666/1-486 CTGTC----CTACCAGGGCGACGAAG-----GT--------CTCCCAGACGAACTCCGTG
gi930068992/1-487 CTGTC----CTACCAGGGCGACAAAG-----GT--------CTCCCAGACGAACTCCGTG
gi930068990/1-487 CTGTC----CTACCAGGGCGACGGAG-----GT--------CTCCCAGACAAACTCCGTG
gi950655841/1-488 CTGTC----CTACCAGGGCGACGAAG-----GT--------CTTCCAAACGAACTCCGTG
gi930068952/1-487 CTGTC----CTACCAGGGCGACGAAG-----GT--------CTCCCAGACGAACTCCGTG
gi58615486/1-470  TGCTC----GTACCAGGGCGATCAGG-----GCGAGTGCCT--------CGAACTCCGTG
gi5616127/1-466   TGCTC----GTACCAAGGTGACCAGGATGCTGC----------------TGAGCTTCGTG
gi82492006/1-464  TTCTCTATCAGAAAC----GATGAAG-----GT--------ATACCACTTGAAATGAGAG
gi262358095/1-470 TTCTCCATCAGAAAC----GATGAAG-----GA--------TTGCCACTTGAAATGAGAG
gi629633272/1-472 TTCTCCATCAGAAAC----GATGAAG-----GA--------TTACCACTCGAAATGAGAG
gi629633282/1-472 TTCTCCATCAGAAAC----GATGAAG-----GA--------TTACCACTCGAAATGAGAG
gi334085683/1-468 TTCTCCATCAGAAAC----GATGAAG-----GT--------TTACCACTCGAAATGAGAG
gi217792440/1-467 TTCTCCTTCAGAAAT----GATGAAG-----GT--------TTACCTCTTGAAATGAGAG
gi498543114/1-395 TTCTCCTTCAGAAAT----GATGAAG-----GT--------TTACCTCTTGAAATGAGAG
gi629633278/1-472 TTCGCTATTAGAAAC----GACGAAG-----GT--------TTACCACTTGAATTAAGAG
gi145370874/1-445 TTCTCTATAAGAAAT----GACGAAG-----GA--------TTACCAGTTGAAATGAGAG
gi311141403/1-440 TTCTCTATAAGAAAT----GACGAAG-----GA--------TTACCAGTTGAAATGAGAG
gi629633284/1-466 TTATCAATTAGGAGT----GATGAAG-----GT--------CTTATCCATGAATTAAGAG
gi629633280/1-466 TTATCTATTAGAAGT----GATGAAG-----GA--------TTAATTCATGAATTAAGAG

gi309253887/1-472 GCGCAAACTACCC-CAACTACGCCATGAA-CG
gi930068978/1-469 GACCAAACTACCC-CAACTACGCAATGA----
gi930068946/1-466 GACCAAACTACCC-CAACTATGCCA-------
gi954036668/1-468 GACCGAACTACCC-TAACTACGCAATG-----
gi930068996/1-469 GAGCCAACTATCC-CAACTATGCAATGA----
gi428274410/1-489 GACCGAACTACCC-CAACTATGCCATA-----
gi954036662/1-94  --------------------------------
gi954036666/1-486 GTCCAAACTATCC-CAACTACGCCATG-----
gi930068992/1-487 GTCCAAACTACCC-TAACTACGCCCTGA----
gi930068990/1-487 GTCCAAACTACCC-CAACTACGCCCTGA----
gi950655841/1-488 GTCCAAACTATCC-AAACTACGCCATGAA---
gi930068952/1-487 GTCCAAACTACCC-CAACTACGCCATGA----
gi58615486/1-470  GCGCAAACTACCCCCAACTACGCNATGA----
gi5616127/1-466   GCGCAAACTACCC-TAACTACGCCATGA----
gi82492006/1-464  GTCCTAACTATCC-TAACTACGC---------
gi262358095/1-470 GGCCAAACTACCC-AAACTACGCAATGAA---
gi629633272/1-472 GACCAAACTATCC-TAACTACGCAATGAA-CG
gi629633282/1-472 GACCAAACTATCC-TAACTACGCAATGAA-TG
gi334085683/1-468 GTCCTAACTATCC-CAACTACGCCATA-----
gi217792440/1-467 GACCTAACTACCC-TAACTACGCAATGAA---
gi498543114/1-395 GACCTAACTATCC-TAACTACGCAATGAAA--
gi629633278/1-472 GACCTAACTATCC-TAACTATGCAATGAA-TG
gi145370874/1-445 GACCAA--------------------------
gi311141403/1-440 G-------------------------------
gi629633284/1-466 GTCCTAATTATCC-TAATTATGCAATGAA-TG
gi629633280/1-466 GGCCTAACTATCC-TAACTATGCAATGAA-TG
```
